# Advanced Bismuth-Based Anode Materials for Efficient Potassium Storage: Structural Features, Storage Mechanisms and Modification Strategies

**DOI:** 10.1007/s40820-024-01641-9

**Published:** 2025-01-31

**Authors:** Yiye Tan, Fanglan Mo, Hongyan Li

**Affiliations:** Department of Materials Science and Engineering, College of Chemistry and Materials Science, Jinan University, Guangzhou, 510632 People’s Republic of China

**Keywords:** Bismuth-based materials, Potassium-ion batteries, Anode, Potassium storage mechanism, Modification strategies

## Abstract

Potassium-ion batteries (PIBs) are considered as a promising energy storage system owing to its abundant potassium resources. As an important part of the battery composition, anode materials play a vital role in the future development of PIBs. Bismuth-based anode materials demonstrate great potential for storing potassium ions (K^+^) due to their layered structure, high theoretical capacity based on the alloying reaction mechanism, and safe operating voltage. However, the large radius of K^+^ inevitably induces severe volume expansion in depotassiation/potassiation, and the sluggish kinetics of K^+^ insertion/extraction limits its further development. Herein, we summarize the strategies used to improve the potassium storage properties of various types of materials and introduce recent advances in the design and fabrication of favorable structural features of bismuth-based materials. Firstly, this review analyzes the structure, working mechanism and advantages and disadvantages of various types of materials for potassium storage. Then, based on this, the manuscript focuses on summarizing modification strategies including structural and morphological design, compositing with other materials, and electrolyte optimization, and elucidating the advantages of various modifications in enhancing the potassium storage performance. Finally, we outline the current challenges of bismuth-based materials in PIBs and put forward some prospects to be verified.

## Highlights

- Various bismuth-based materials used in potassium-ion batteries (PIBs) anode are classified and overviewed, and the structure and potassium storage mechanism of various materials are discussed.
- The advantages and challenges of different PIBs anode materials are pointed out, and the existing modification strategies to improve potassium storage are summarized.
- The promising research directions of bismuth-based anode materials are proposed.

## Graphical abstract

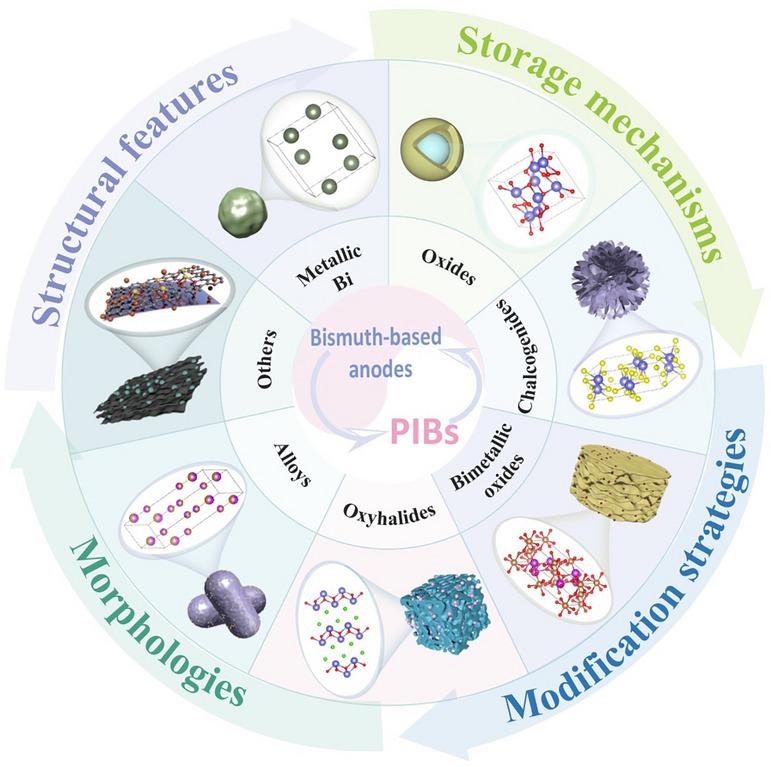

## Introduction

There is a growing demand for energy storage devices for environmental and developmental reasons [1]. Over the past few decades, lithium-ion batteries (LIBs) have dominated the market for portable electronic devices and electric vehicles due to their high energy density, long cycle life and environmental friendliness [2]. However, the scarcity and uneven distribution of lithium resources hinder the sustainable development of LIBs [3]. Sodium-ion batteries (SIBs) and potassium-ion batteries (PIBs) have attracted extensive attention due to the similar physicochemical properties to LIBs and abundant resources [4]. It is worth noting that three advantages of PIBs over SIBs have been demonstrated: (1) The redox potential of K/K^+^ is − 2.93 V (vs. SHE), which is closer to Li/Li^+^ (− 3.04 V) than Na/Na^+^ (− 2.71 V), so PIBs may exhibit higher voltage. This could result in a wider voltage window and higher energy density for PIBs [5]. (2) Another advantage of PIBs is the low Lewis acidity of K^+^ in organic solvents, which leads to small solvated K^+^ [6] and low desolvation energy [7]. Thus, the transmission conductivity and quantity of solvated K^+^ are much greater than that of Li^+^ and Na^+^. (3) K^+^ can be electrochemically embedded in and deintercalated from the interlaminar space of graphite, whereas the insertion and extraction of Na^+^ can only occur in the co-intercalation of ether molecules. As a result, the specific capacity of SIBs in graphite (110 mAh g^−1^) is much lower than that of PIBs (279 mAh g^−1^) [2].

However, the development of PIBs currently faces some challenges. Firstly, the large ionic radius of the K^+^ results in a large structural change of the electrode during the potassiation/depotassiation process [8]. Secondly, the slow kinetics of K^+^ limits the rate performance of PIBs. Finally, metallic potassium directly used as an anode has potential safety hazard [9]. In 2015, commercial graphite was used in PIBs and measured that the reversible capacity reached 273 mAh g^−1^. However, the graphite expansion of 61% is observed during the potassium storage process, which results in rapid capacity decay and low-rate performance of PIBs [10]. Thus, a lot of works have been devoted to developing more advanced and efficient anode materials to effectively improve the performance of PIBs, such as carbonaceous materials, conversion materials, alloying materials, metal oxides and organic materials [11].

Among these potential materials, bismuth metals and bismuth-based materials are widely studied owing to their ideal theoretical capacity, safe operating voltage and environmental friendliness [12]. Bismuth can provide a specific capacity of 385 mAh g^−1^ with the formation of K_3_Bi with K, which is much higher than graphitic carbon. In addition, Bi has large lattice fringes along the *c*-axis, which effectively promotes K^+^ insertion. At the same time, bismuth-based materials have high electrical conductivity and easiness for preparing nanostructured anode, which has far-reaching significance for improving the performance of PIBs [13]. Based on the above advantages, the slow kinetics of K^+^ is accelerated, which is conducive to improving the excellent rate performance of PIBs. Besides, the polarization effect of the electrode during charging/discharging process will be alleviated, which will significantly improve the Coulombic efficiency of PIBs. Therefore, not only the safety problem of PIBs has been effectively solved, but also the low and safe operating voltage of bismuth-based materials is beneficial to increase the energy density of PIBs [14]. However, the bismuth-based materials based on alloying reactions will inevitably cause volume expansion (406%) during potassium storage, leading to aggregation and pulverization of the active material [15]. Combined with an unstable reaction interface, the battery exhibits rapid capacity degradation, short cycle life and poor rate capability [16]. These greatly limit the development of bismuth-based materials in PIBs.

A range of strategies have been proposed to address the above issues, and significant progress has been made. The strategy mainly includes the design of nanostructures and morphologies [17], the construction of heterogeneous structures [18], the composite with carbonaceous materials [19] and the optimization of electrolytes. There are many works summarizing the recent progress of bismuth-based materials applied in PIBs anode. However, no work has been done to summarize the modification strategies of different types of bismuth-based materials based on their characteristics, potassium storage mechanisms and advantages and problems faced in potassium storage.

Herein, we summarize the recent research on bismuth-based anode materials for PIBs, including bismuth metals, bismuth-based oxides, chalcogenides, alloys, bimetallic oxides, oxyhalide and their composites. In order to offer a comprehensive and in-depth overview of the bismuth-based materials, we summarize the strategies used to improve the potassium storage properties of various types of materials, and introduce recent advances in the design and fabrication of favorable structural features of bismuth-based materials. Firstly, the structures and working mechanism of the various types of materials are presented, highlighting the advantages and disadvantages in potassium storage, as well as the importance of characterization techniques in the study of potassium storage mechanisms. Secondly, in response to the problems faced so far, the review focuses on summarizing modification strategies including structural and morphological design, compositing with other materials, and electrolyte optimization, and elucidating the advantages of various modifications in enhancing the potassium storage performance. Finally, the challenges as well as the prospects for high-performance bismuth-based anode materials for PIBs are proposed. We believe that this review will help researchers understand the basic knowledge of bismuth-based materials in PIBs and provide guidance and foundation for the design of efficient and advanced anode materials.

## Bismuth-Based Materials in PIBs

### Metallic Bismuth and Its Composites

Metallic bismuth as an environmentally friendly transition metal is considered as a potential anode material for PIBs due to its favorable layered crystal structure, high theoretical capacity and environmental friendliness. As a kind of alloying material, the bismuth metal stores potassium by alloying with K^+^ in PIBs. The final product (K_3_Bi) formed after alloying has a high theoretical capacity of 385 mAh g^−1^ [20].

**Fig. 1:**
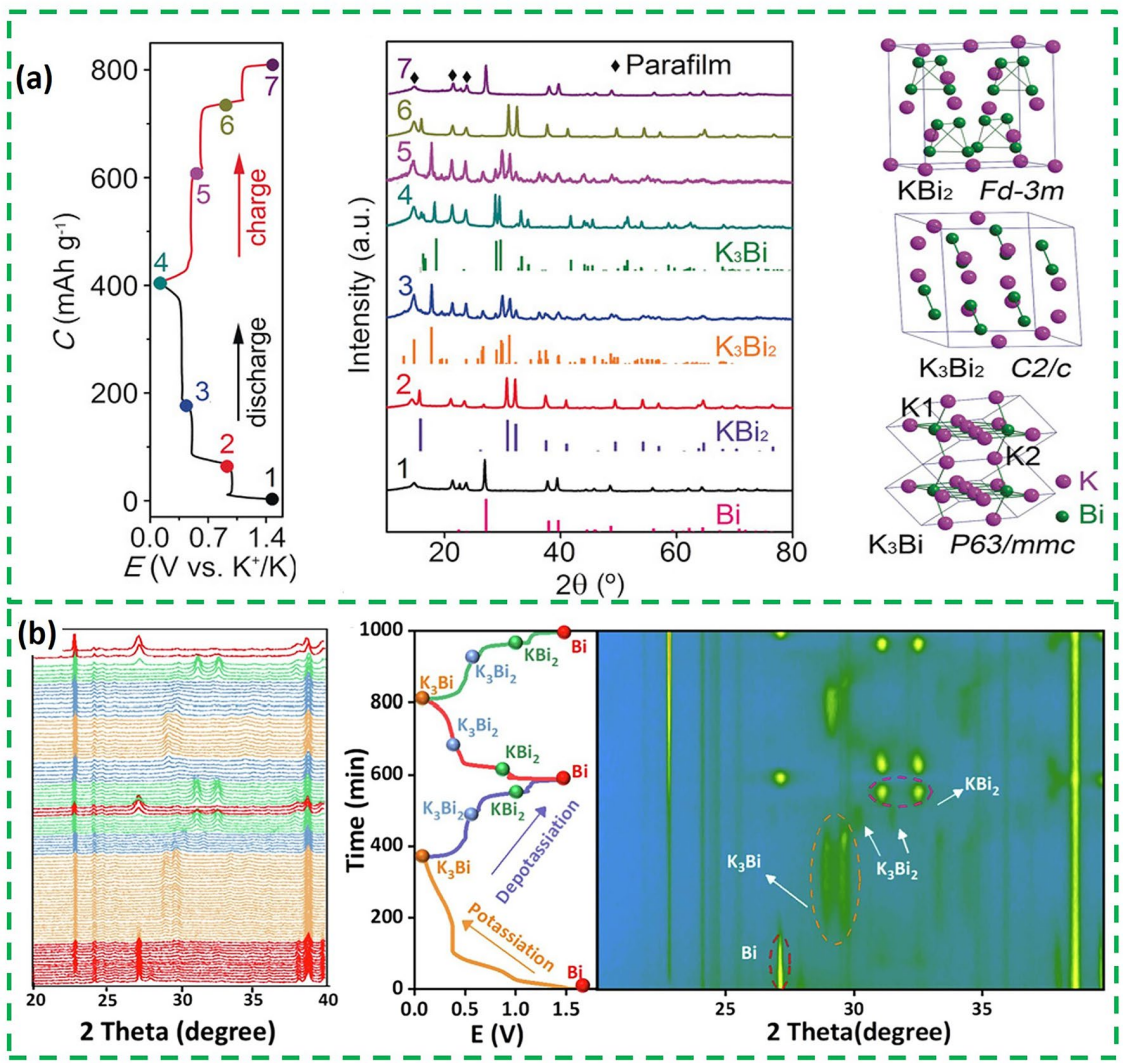
**a** XRD patterns of Bi [22]. Copyright 2023, Wiley–VCH GmbH. **b** In situ XRD of Bi NPs/NPC [19]. Copyright 2022, Elsevier B.V

So far, a variety of characterization techniques have been used to reveal the alloy-type potassium storage mechanism of bismuth metal [21]. In 2018, a study reveals the phase changes during potassium storage process of commercial bismuth particles by ex situ X-ray diffraction (XRD) [22]. During the discharge process, the diffraction peaks of KBi_2_, K_3_Bi_2_ and K_3_Bi were observed at 0.9, 0.45 and 0.01 V, respectively (Fig. 1a). A reverse conversion is observed during the subsequent charging. It proved that there are three reversible two-phase reactions between Bi and K^+^ during the potassiation/depotassiation process; the specific reaction process is as follows:

1 $${\text{2Bi }} + {\text{ K}}^{ + } + {\text{ e}}^{ - } \to {\text{ KBi}}_{{2}}$$

2 $${\text{KBi}}_{{2}} + {\text{ 2K}}^{ + } + {\text{ 2e}}^{ - } \to {\text{ K}}_{{3}} {\text{Bi}}_{{2}}$$

3 $${\text{K}}_{{3}} {\text{Bi}}_{{2}} + {\text{ 3K}}^{ + } + {\text{ 3e}}^{ - } \to {\text{ 2K}}_{{3}} {\text{Bi}}$$

Different to the typical three-step alloying reaction described above, the in situ XRD (Fig. 1b) of Bi NPs/NPC (Bi nanoparticles located in the *N*-doped porous carbon network) revealed that the alloying reaction of Bi and K^+^ directly forms K_3_Bi during the first discharge, which is different to the three-step alloying of the subsequent discharge process [19]. Furthermore, in addition to the three-step and one-step alloying reactions described above, Bi sometimes stores potassium by two-step alloying, i.e., there is no intermediate K_3_Bi_2_. The specific process is Bi ↔ KBi_2_ ↔ K_3_Bi [23]. This unique difference of the three alloying processes may be attributed to the effect of the microstructure of Bi on surface/interface reactions and phase transitions.

The research of Bi in PIBs is based on its high theoretical capacity and alloying potassium storage mechanism. However, metal Bi still faces the following three problems:

1. According to the mechanism analysis, metal Bi experiences significant volume changes during repeated potassiation/depotassiation processes, which directly leads to the pulverization of the anode material and then its removal from the current collector and a sharp decay of capacity.
2. The electronic conductivity of Bi is 7.75 × 10^3^ S m^−1^ [24]. The low inherent conductivity directly affects the transport of K^+^, which make battery shows poor rate performance [25].
3. Unstable solid electrolyte interfaces (SEI). At present, many strategies have been studied to form a stable SEI film at the bismuth-based anode, including the selection of a suitable electrolyte and the construction of a stable electrode structure [26].

**Fig. 2:**
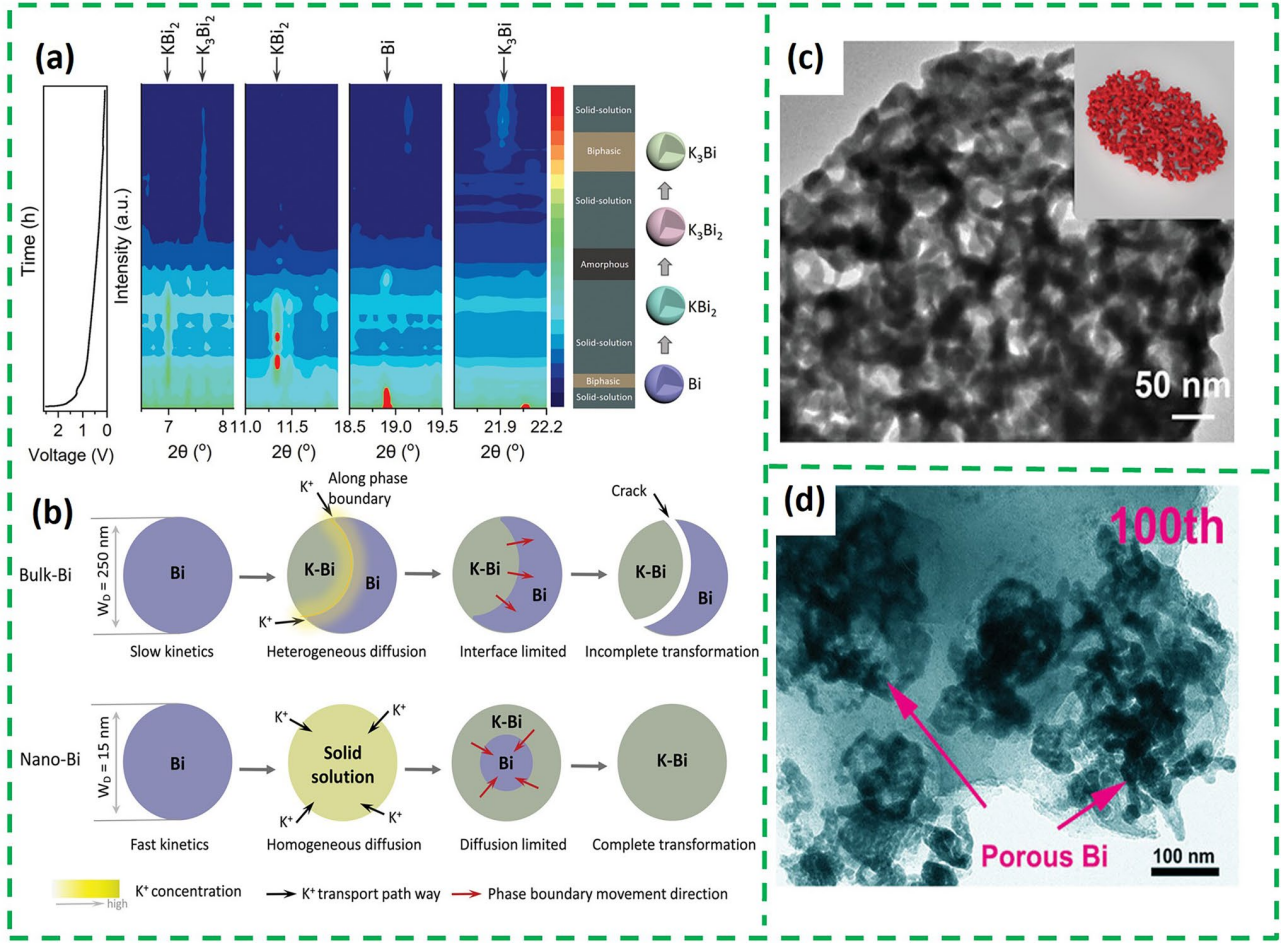
**a** Voltage profile and corresponding contour plot of the in situ SXRD pattern of nano-Bi during the initial potassiation process. **b** Schematic illustration of the different potassiation mechanisms for bulk Bi and nano-Bi [28]. Copyright 2022, Wiley–VCH GmbH. **c** Post-cycle TEM images of the 2D-Bi, the inset image is the schematic illustration of Bi in 2D-Bi [29]. Copyright 2021, Wiley–VCH GmbH. **d** TEM images of Bi NSs/NCNs after 100th cycles at 1 A g^−1^ [34]. Copyright 2021, Wiley–VCH GmbH

The design of nanostructures can effectively shorten the electron/ion transport path to improve the reaction kinetics of PIBs [27]. In addition, the spaces between the small size nanomaterials and within nanostructures can alleviate mechanical stresses caused by volume expansion of the active material. The size-dependent phase transition mechanism of nanostructures in inhibiting mechanical deterioration during potassicization/depotassicization is revealed by in situ XRD [28]. For bulk potassium, individual particles undergo a non-homogeneous two-phase transition with anisotropic expansion, thus gradually increasing the stress during initial cycling, and then leads to mechanical fracture (Fig. 2b), which manifests itself as an abrupt change in the voltage plateau during cycling. However, nano-sized Bi with a solid-solution single-phase reaction at non-equilibrium states (Fig. 2a) produces anisotropic expansion stress due to the small size, without mechanical fracture (Fig. 2b). Based on this, different synthesis methods were used to control the size of bismuth and prepare zero-dimensional to three-dimensional nanobismuth, respectively [29]. The 2D-Bi is transformed into a continuous porous bismuth nanoligament after the cycle retaining the shape of the original nanosheet (Fig. 2c). The special nanostructure can buffer the volume change during the potassium storage process and shorten the diffusion path of K^+^.

However, it is worth noting that the substantial reduction in size means an obvious increase in the specific surface area and interface between the active material and the electrolyte. On the one hand, large specific surface area tends to consume a large quantity of electrolyte and reduce the Coulomb efficiency. On the other hand, excessive electrode/electrolyte interface leads to serious side reactions, which form irreversible SEI films. Therefore, the reasonable bismuth particle size is crucial. Since it is difficult to realize efficient potassium storage by single size control, on this basis combination of Bi nanoparticles with other conductive substances, such as one-dimensional (1D) nanorods, two-dimensional (2D) nanomaterials or three-dimensional (3D) networks, can effectively stabilize the electrode structure and improve the cyclic stability of PIBs. 1D material can not only improve the conductivity and mechanical strength of Bi anode, but also can be interwoven into a network structure to shorten the transport path of K^+^ [30]. Moreover, the large gap between one-dimensional materials is not only conducive to the full contact between the material and the electrolyte, but also can effectively alleviate the volume expansion of Bi particles, thus effectively improving the cycle stability of electrode [31].

Compared with 1D materials, 2D materials have the advantages of shorter ion diffusion distance and larger specific surface area, which may more effectively improve the potassium storage performance of the anode. However, the 2D nanosheets have a serious restacking tendency, which hinders electron/ion transport and slows down the kinetics of the battery. The combination of Bi nanoparticles with highly conductive 2D materials (such as graphene) not only effectively mitigates the volume expansion of Bi nanoparticles and improves charge transfer, but also eliminates the restacking tendency of 2D materials. In addition, some methods (such as the reduction of Bi_2_O_3_ precursor) can also be used to create void space in the 2D composites, which can further improve the kinetic and volume expansion problems [32].

3D porous carbon materials are usually composed of continuously interconnected network structures, with enormous specific surface area and excellent electrical conductivity [33]. Therefore, 3D porous carbon materials are often used as an ideal buffer framework to alleviate the volume expansion of Bi nanoparticles, promote electrolyte penetration, accelerate electron/ion transport and enhance electrode stability. Bi nanospheres (~ 149 nm) embedded in 3D N-riched carbon nanonetworks (Bi NSs/NCNs) were synthesized by electrospinning technology [34]. As shown in Fig. 2d, the NCNs framework enabled the porous nanostructures Bi to maintain the integrity of their interconnections even after 100 cycles. Based on interconnected porous nanostructures, Bi NSs/NCNs obtained enhanced rate performance (489.3 mAh g^−1^ at 50 A g^−1^).

However, a single strategy is often difficult to meet the performance needs of the battery. In addition to the design of the above three nanostructures, the combination of the two strategies of designing nanostructures and composite carbonaceous materials to form bismuth-based hybrid structures can give full play to the advantages of both at the same time. Recently, Bi@C nanospheres have been loaded onto 3D porous graphene (GR) nanosheets to form composite Bi@C@GR [35]. The Bi nanoparticles form a core–shell structure with the carbon layer, which protects the volume variation of Bi nanoparticles and improves the rate performance and structural stability of the electrode. Besides, graphene can improve the electrical conductivity, enlarges specific surface area and increases the mechanical stress of the material.

**Fig. 3:**
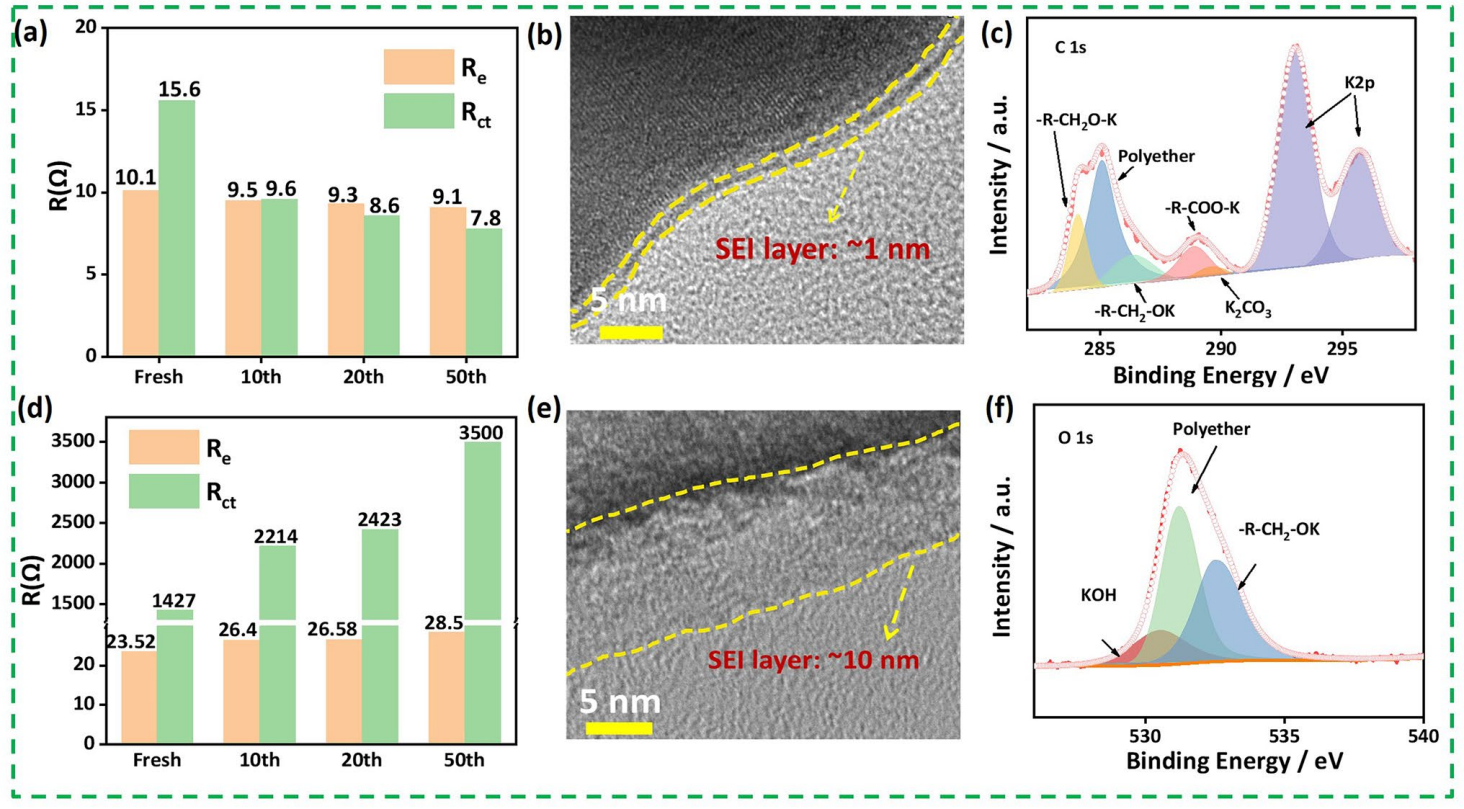
**a, d** Corresponding *R*_e_ and *R*_ct_ values after cycles in DME and EC/DEC-based electrolytes. **b, e** HRTEM image of SEI after cycle in DME and EC/DEC-based eletrolytes. **c, f** Ex situ XPS of C 1*s* and O 1*s* of Bi NPs/NPC in DME-based electrolyte after cycles [19]. Copyright 2022, Elsevier B.V

Another approach to improve the electrochemical performance of bismuth-based anodes is replacing carbonate electrolyte with ether-based electrolyte. Ether electrolytes can help maintain the stability of the electrode interface due to their low activity in the low-voltage (reducing) environment [36]. It is worth noting that the initial Coulomb efficiency (ICE) of a half-cell is defined as the capacity of the first charge (depotassiumization) divided by the capacity of the first discharge (potassiumization). During the first discharge, an irreversible side reaction occurs on the surface of the anode, resulting in irreversible K^+^ loss, which is manifested as ICE less than 100%. The irreversible decomposition of the electrolyte as a side reaction also affects the ICE. A work reported that the ICE of Bi NPs/NPC in EC/DEC and DME was 52.8% and 76.05%, respectively [19], and the DME electrolyte-based electrodes exhibited excellent cycling stability and multiplicity performance. It was found through ex situ EIS that (Fig. 3a, d) the *R*_CT_ decreases as the cycle progresses and remains stable after 50 cycles in DME. Besides, the HRTEM shows that compared to EC/DEC-based electrolytes, a thinner and homogeneous SEI film can be observed in DME-based electrolytes (Fig. 3b, e). Furthermore, the ex situ XPS revealed that the SEI in DME consists of flexible and elastic organic polyethers and oligomers (-R-C_2_HO-K) with strong binding affinity (Fig. 3c, f). So, the homogeneous SEI with elastic and thin nature formed in the DME-based electrolyte allowed the electrodes to remain structurally stable during repeated potassiation/depotassiation processes. This provides a reference for the selection of bismuth-based anode electrolytes based on alloy-type reactive potassium storage.

As shown in Table 1, many bismuth metals and its composites have been developed in recent years for application in the anode of PIBs. At present, the preparation methods of metallic bismuth and its composites mainly include pyrolysis and carbothermal reduction method, thermal treatment, electrospinning method, hydrothermal method, ultrasonication-assisted electrochemical exfoliation method and etching method. Overall, the above strategies can effectively improve the main problems currently faced by metal Bi. However, it is worth noting that although the composite of metal Bi with other conductive materials can effectively improve the conductivity of the material, the intrinsic conductivity of the metal Bi itself will not be changed, and therefore it must be designed from an atomic point of view, such as doping or defects, in order to improve the conductivity and thus enhance the electrochemical properties of the material.

**Table 1:** Performance comparison of metallic bismuth anode

Metallic bismuth anode of PIBs					
Materials	Rate capability	Cycle capability	ICE (%)		Refs
BiND/G	320 mAh g^–1^ at 0.1 A g^–1^; 165.3 mAh g^–1^ at 10 A g^–1^	180 mAh g^–1^ after 500 cycles at 10 A g^–1^	78		[37]
Nano-sized Bi	125 mAh g^–1^ at 500 mA g^–1^	100 cycles at 100 mA g^–1^	–		[38]
C@DSBC	340 mAh g^–1^ at 40 mA g^–1^; 222 mAh g^–1^ at 800 mA g^–1^	200 cycles at 200 mA g^–1^	52		[39]
Bi@N-CT	316 mAh g^–1^ at 1 C; 297 mAh g^–1^ at 20 C	266 mAh g^–1^ after 1000 cycles at 10 C	–		[40]
Bi@N–C	300 mAh g^–1^ at 1 A g^–1^; 178 mAh g^–1^ at 100 A g^–1^	235 mAh g^–1^ after 2000 cycles at 10 A g^–1^	36.5		[41]
2D-Bi	395 mAh g^–1^ at 1 A g^–1^; 345 mAh g^–1^ at 30 A g^–1^	344 mAh g^–1^ after 750 cycles at 10 A g^–1^	89.2		[29]
UCF@CNs@BiN	430 mAh g^–1^ at 100 mA g^–1^; 140 mAh g^–1^ at 1.0 A g^–1^	327 mAh g^–1^ after 600 cycles at 100 mA g^–1^	–		[42]
Bi/PPy/CNT	361 mAh g^–1^ at 0.1 A g^–1^; 267.7 mAh g^–1^ at 3.2 A g^–1^	195.7 mAh g^–1^ after 600 cycles at 1 A g^–1^	37		[43]
Bi@NS-C	404.2 mAh g^–1^ at 0.2 A g^–1^; 399.5 mAh g^–1^ at 20 A g^–1^	350.3 mAh g^–1^ after 800 cycles at 1 A g^–1^	80		[31]
Bi@C@GR	386 mAh g^–1^ at 0.1 A g^–1^; 68 mAh g^–1^ at 1.6 A g^–1^	200 mAh g^–1^ after 70 cycles at 0.1 A g^–1^	46		[35]
Bi@N-CNCs	334.3 mAh g^–1^ at 0.5 A g^–1^; 235.5 mAh g^–1^ at 10 A g^–1^	224 mAh g^–1^ after 1200 cycles at 5 A g^–1^	78		[23]
Bulk Bi	371.4 mAh g^–1^ at 2 C; 321.9 mAh g^–1^ at 3 C	322.7 mAh g^–1^ after 300 cycles at 2 C	87.2		[22]
Bi NSs/NCNs	565.2 mAh g^–1^ at 1 A g^–1^; 489.3 mAh g^–1^ at 50 A g^–1^	457.8 mAh g^–1^ after 2000 cycles at 10 A g^–1^	71.2		[34]
Bi@N–C	328 mAh g^–1^ at 1 A g^–1^; 152 mAh g^–1^ at 100 A g^–1^	203 mAh g^–1^ after 1000 cycles at 10 A g^–1^	43		[32]
FBNs	423 mAh g^–1^ at 2.5 A g^–1^; 227 mAh g^–1^ at 15 A g^–1^	200 mAh g^–1^ after 2500 cycles at 20 A g^–1^	–		[44]
Bi NPs/NPC	377 mAh g^–1^ at 0.5 A g^–1^; 362.4 mAh g^–1^ at 20 A g^–1^	277.5 mAh g^–1^ after 1600 cycles at 5 A g^–1^	75.89		[19]
SPB@NC	404.9 mAh g^–1^ at 0.5 A g^–1^; 276.5 mAh g^–1^ at 30 A g^–1^	299.3 mAh g^–1^ after 2000 cycles at 5 A g^–1^	79.89		[45]
2D Bi@NOC	392.9 mAh g^–1^ at 1 A g^–1^; 220.6mAh g^–1^ at 50 A g^–1^	341.7 mAh g^–1^ after 1000 cycles at 10A g^–1^	–		[46]
HD-Bi@G	365.3 mAh g^–1^ at 0.5 A g^–1^; 296.8 mAh g^–1^ at 20 A g^–1^	349.9 mAh g^–1^ after 300 cycles at 1 A g^–1^	90.8		[47]
BC-NCS	305 mAh g^–1^ at 0.1 A g^–1^; 118 mAh g^–1^ at 5 A g^–1^	275 mAh g^–1^ after 100 cycles at 0.1 A g^–1^	31.3		[48]
Bi@Void ⊂ CNF	388.8 mAh g^–1^ at 0.05 A g^–1^; 64.9 mAh g^–1^ at 10 A g^–1^	171.6 mAh g^–1^ after 3000 cycles at 2 A g^–1^	35		[49]
Nano-Bi	250 mAh g^–1^ at 0.05 A g^–1^; 52 mAh g^–1^ at 2 A g^–1^	260 mAh g^–1^ after 100 cycles at 0.05 A g^–1^	39.5		[28]

### Bismuth Oxide and Their Composites

As a semiconductor compound, bismuth oxide (Bi_2_O_3_) is widely used in the fields of photocatalysis and energy storage due to its high stability and environmental friendliness. Owing to its high specific capacity of 690 mAh g^−1^, Bi_2_O_3_ is considered as a potential anode material for rechargeable batteries [50]. The crystal structure of Bi_2_O_3_ is dominated by monoclinic crystal system, with large interstorey spaces and a stable main structure, in which the void between two successive ion layers can provide a lot of space for ion embedding, which is conducive to potassium storage.

**Fig. 4:**
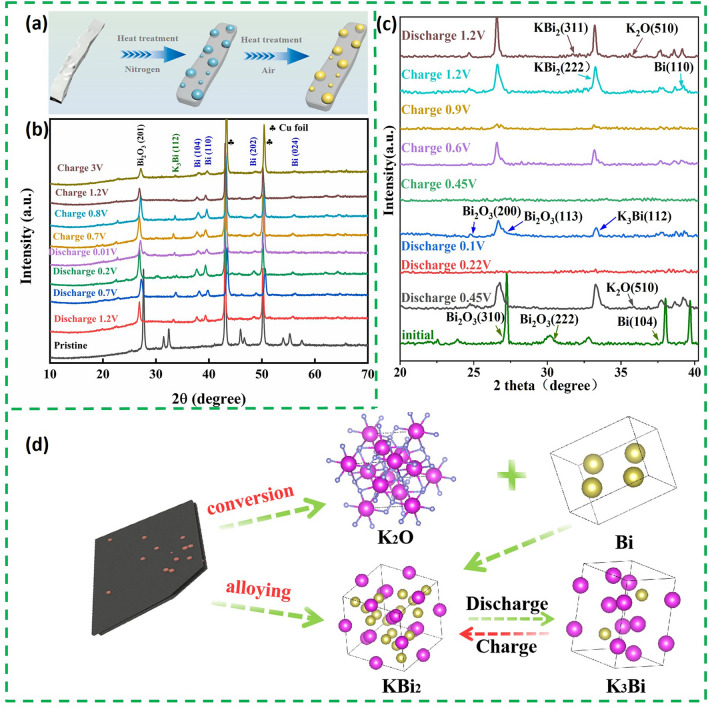
**a** Schematic illustration of the fabrication of Bi_2_O_3_@C. **b** Charge and discharge curves and ex situ XRD patterns of the Bi_2_O_3_@C electrodes at different states [51]. Copyright 2022, Elsevier B.V. **c** Ex situ XRD of Bi/Bi_2_O_3_@CN. **d** Schematic illustration of K^+^ storage mechanism of Bi/Bi_2_O_3_@CN [52]. Copyright 2024, Elsevier Inc

The potassium storage mechanism of Bi_2_O_3_-based composite consists of conversion reaction and alloying reaction. The advantage of this dual potassium storage mechanism is that the actual capacity of Bi_2_O_3_-based composite is derived from two parts, which means that it can achieve a higher capacity than metallic Bi in most cases, which has only one mode of potassium storage. So far, in situ TEM and ex situ XRD techniques have been used to reveal the potassium storage mechanism of Bi_2_O_3_. The ex situ XRD of Bi_2_O_3_@C (the synthesis schematic is shown in Fig. 4a) and Bi/Bi_2_O_3_@CN (Bi/Bi_2_O_3_ nanoparticles assembled on *N*-doped carbon sheets) are shown in Fig. 4b, c, respectively [51, 52]. The peaks of Bi, K_2_O and K_3_Bi can be observed in both ex situ XRD patterns. The difference is that Bi/Bi_2_O_3_@CN can detect a peak for KBi_2_, which is not present in the Bi_2_O_3_@C. All these indicate that Bi_2_O_3_ undergoes a conversion reaction with K^+^ and then followed by alloying process. It is just that the intermediates of the alloying process vary from material to material. The corresponding schematic diagram is shown in Fig. 4d.

Currently, the potassium storage performance of Bi_2_O_3_ is mainly limited by its poor electrical conductivity and severe volume expansion due to conversion and alloying reactions during potassium storage. Many Bi_2_O_3_ composites have been reported to improve the performance of PIBs. Currently, the main strategies are compositing with carbonaceous materials, constructing heterogeneous structures and building buffer layers.

**Fig. 5:**
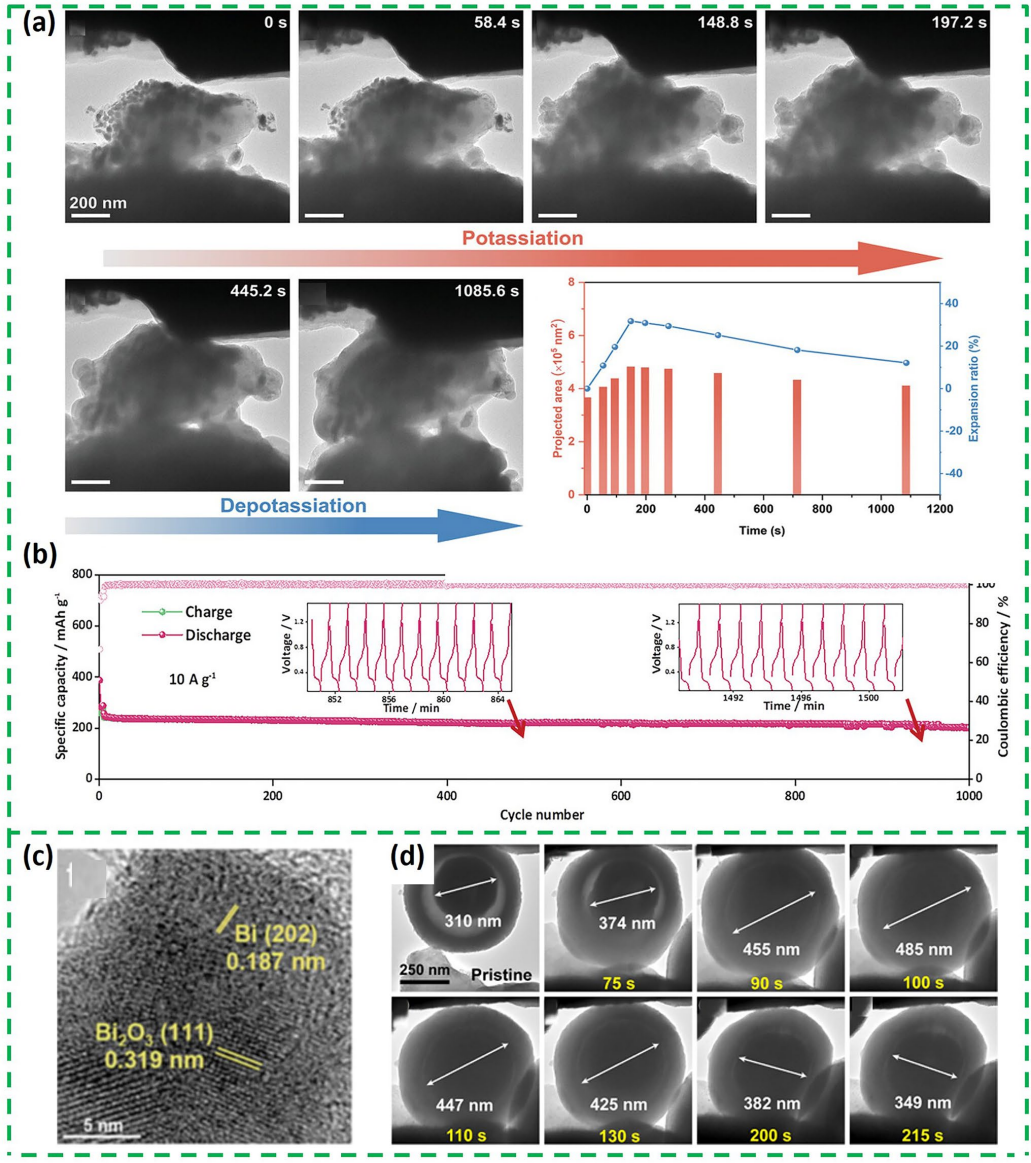
**a** In situ TEM observation of the morphology evolution during live potassiation/depotassiation process and the corresponding size and projected area evolution during initial cycle for Bi/Bi_2_O_3_ NDs@CSs. **b** Cycling properties of 10 A g^−1^ [25]. Copyright 2023, Wiley‐VCH GmbH. **c** HRTEM images of the Bi/Bi_2_O_3_-C-900 [54]. Copyright 2022, Elsevier Inc. **d** In situ TEM images of a single y-Bi_2_O_3_@TiO_2_ sphere during potassiation and depotassiation [55]. Copyright 2022, Science China Press

At present, introducing carbonaceous materials or constructing Bi_2_O_3_@C structures is the most common methods to improve the electrochemical properties of Bi_2_O_3_. Carbonaceous materials can not only alleviate the volume expansion of Bi_2_O_3_ and stabilize the material structure, but also provide a large number of defects and active sites and shorten the electron/ion transport channel [53]. A core–shell-structured Bi/Bi_2_O_3_@CSs composite was designed by solvothermal and carbothermal methods to address the structural stability of Bi_2_O_3_ [25]. The modification of the outer layer of the porous carbon sheet not only introduced active sites, but also accelerated the electronic conduction of K/K^+^ and enhanced the structural stability. Based on the synergistic effect of Bi/Bi_2_O_3_ nanoparticles and carbon nanosheets, the overall structure of Bi/Bi_2_O_3_ NDs@CSs can be still well maintained without any visible mechanical damages during the whole potassiation/depotassiation process (Fig. 5a) and exhibited high multiplicity capacity and stable cycling performance in PIBs (Fig. 5b, more than 200 mAh g^−1^ in 10 A g^−1^ after 1000 cycles). This work provides a foundation for the design of bismuth-based anode materials with strain-relaxation function. At the same time, it provides a valuable reference for the composite materials of bismuth metal, bismuth-based oxide and carbon composites, and paves the way for the commercialization of bismuth-based potassium electrodes in the future.

Besides, constructing heterogeneous structures of various components can improve the reaction kinetics by the action of built-in electric fields. A study pyrolyzed Bi–MOF with an accordion-like structure to prepare a plate-like Bi/Bi_2_O_3_-C-900 heterostructure (Fig. 5c) [54]. This heterogeneous structure resulted in a large number of heterogeneous interfaces between the Bi alkali particles and the carbon skeleton, which facilitated interfacial charge transfer.

In addition to composite with carbonaceous materials, using non-carbonaceous materials as a buffer layer for volume change is an effective strategy that deserves further exploration. Bi_2_O_3_ structures with large internal voids can also be constructed to buffer volume expansion. Recently, core–shell structures of *y*-Bi_2_O_3_@TiO_2_ were designed as an anode material of PIBs [55]. The flexible TiO_2_ shell exhibits elasticity during charging and discharging to confine Bi_2_O_3_ within the shell, and the void between the core and shell can effectively accommodate the volume change of Bi_2_O_3_ during cycling (Fig. 5d). As shown in Table 2, in addition to these above works, there are also many Bi_2_O_3_ composites applied to PIBs.

**Table 2:** Performance comparison of bismuth oxide

Bismuth oxide anode of PIBs					
Materials	Rate capability	Cycle capability	ICE (%)		Refs
Bi/Bi_2_O_3_-C	426.0 mAh g^–1^ at 0.05 A g^–1^; 82.7 mAh g^–1^ at 1 A g^–1^	81.5 mAh g^–1^ after 900 cycles at 0.5 A g^–1^	81		[54]
Bi/Bi_2_O_3_ NDs@CSs	149.3 mAh g^–1^ at 60 A g^–1^;	252.7 mAh g^–1^ after 1800 cycles at 5 A g^–1^	–		[25]
Bi_2_O_3_@C	350 mAh g^–1^ at 50 mA g^–1^; 188 mAh g^–1^ at 1000 mA g^–1^	314 mAh g^–1^ after 100 cycles at 50 mA g^–1^	55		[51]
Bi_2_O_3_@TiO_2_	373.9 mAh g^–1^ at 100 mA g^–1^; 139.1 mAh g^–1^ at 2 A g^–1^	216.8 mAh g^–1^ after 500 cycles at 0.5 A g^–1^	64.8		[55]
Bi/Bi_2_O_3_-CN	362.8 mAh g^–1^ at 0.2 A g^–1^; 234.7 mAh g^–1^ at 20 A g^–1^	162.4 mAh g^–1^ after 1500 cycles at 2 A g^–1^	–		[52]
Bi/Bi_2_O_3_/G-50	636.1 mAh g^–1^ at 0.3 A g^–1^; 294.1 mAh g^–1^ at 0.8 A g^–1^	179.1 mAh g^–1^ after 100 cycles at 0.8 A g^–1^	74.2		[56]

The above strategies can effectively improve the problems of poor electrical conductivity and volume expansion faced by Bi_2_O_3_. However, further studies on the preparation methods and conditions of Bi_2_O_3_ and its composites, such as hydrothermal method, solvothermal process, nanocasting and chemical polymerization, electrospinning, etching and template methods, are needed to construct more suitable structures and buffer layers. In addition, Bi_2_O_3_ and carbonaceous materials are both layered materials, and starting from interlayer engineering and defects to improve the problems of volume expansion and low electrical conductivity is also an important aspect of Bi_2_O_3_ and its composites that needs to be studied. Besides, it is worth noting that amorphous Bi_2_O_3_ binds more tightly to carbonaceous materials compared to alloyed materials. Therefore, the use of Bi_2_O_3_ as an alloy coating to prevent the shedding of active materials during cycling is also an important aspect of future research on Bi_2_O_3_.

### Bismuth Chalcogenides (Bi_a_X_b_ (***X*** = S, Se, Te)) and Their Composites

In general, the introduction of suitable inactive anions can act as a bulk buffer, thereby improving the cycling properties of metal alloys [57]. Combining element Bi with chalcogens elements (S, Se and Te) has attracted extensive attention in the field of energy storage due to its efficient energy storage mechanism, low preparation cost, environmental friendliness and high structural stability [58]. The synthetic methods of bismuth chalcogenides (Bi_a_X_b_ (*X* = S, Se, Te)) and their composites involve electrostatic self-assembly, sono-chemical approach, high-energy mechanical milling, solvothermal method, cation exchange process, freeze-drying method and hydrothermal method. The active substances of Bi_a_X_b_ mainly include bismuth sulfide, bismuth selenide and bismuth telluride. Similar to Bi_2_O_3_, the potassium storage mechanism of Bi_a_X_b_ is the combination of conversion and alloying reaction. This storage mechanism not only provides low operating voltages that benefit from the alloying reaction, but also effectively mitigates the large volume expansion by utilizing the transformation products as a buffer for the alloying/dealloying behavior. The relevant works are listed in Table 3.

**Table 3:** Performance comparison of bismuth chalcogenides

Bismuth chalcogenides anode of PIBs					
Materials	Rate capability	Cycle capability		ICE (%)	Refs
NGBS	704.3 mAh g^–1^ at 2 A g^–1^; 327.8 mAh g^–1^ at 10 A g^–1^	427 mAh g^–1^ after 100 cycles at 5 A g^–1^		–	[59]
Bi_2_S_3_/Bi_2_Se_3_ vdWHs	604 mAh g^–1^ at 0.05 A g^–1^; 89 mAh g^–1^ at 3.5 A g^–1^	1000 cycles at 500 mA g^–1^		80	[69]
Bi_2_S_3_@RGO	538 mAh g^–1^ at 200 mA g^–1^;	237 mAh g^–1^ after 300 cycles at 2 A g^–1^		–	[60]
Bi_2_S_3_/rGO	377.1 mAh g^–1^ at 50 mA g^–1^; 260.6 mAh g^–1^ at 300 mA g^–1^	206.92 mAh g^–1^ after 1200 cycles at 0.1 A g^–1^		97.43	[70]
Bi_2_S_3_@SC	626 mAh g^–1^ at 50 mA g^–1^; 268.9 mAh g^–1^ at 1 A g^–1^	250 cycles at 0.5 A g^–1^		62.3	[61]
BMS@NC	300.2 mAh g^–1^ at 3 A g^–1^	382.8 mAh g^–1^ after 100 cycles at 0.1 A g^–1^		78.72	[71]
Bi_2_S_3_@rGO	586 mAh g^–1^ at 0.1 A g^–1^	410mAh g^–1^ after 1000 cycles at 0.1 A g^–1^		–	[72]
Bi_2_Se_3_@C	684 mAh g^–1^ at 100 mA g^–1^; 304 mAh g^–1^ at 2 A g^–1^	543 mAh g^–1^ after 5000 cycles at 1 A g^–1^		68	[73]
Bi_2_Se_3_@NC@rGO	612.0 mAh g^–1^ at 100 mA g^–1^; 101.6 mAh g^–1^ at 5 A g^–1^	113.5 mAh g^–1^ after 1000 cycles at 5 A g^–1^		70.3	[63]
Bi_2_Se_3_@C	568 mAh g^–1^ at 50 mA g^–1^; 210 mAh g^–1^ at 2 A g^–1^	214 mAh g^–1^ after 1000 cycles at 1 A g^–1^		74	[74]
Bi@Bi_4_Se_3_@C	425 mAh g^–1^ at 200 mA g^–1^; 360 mAh g^–1^ at 4 A g^–1^	169.1 mAh g^–1^ after 100 cycles at 0.2 A g^–1^		58.1	[64]
Bi_2_Se_3_@NC	271.8 mAh g^–1^ at 50 mA g^–1^; 194.5 mAh g^–1^ at 0.3 A g^–1^	130.5 mAh g^–1^ after 2000 cycles at 1 A g^–1^		72.58	[65]
Bi/Bi_3_Se_4_@CNR	393.0 mAh g^–1^ at 500 mA g^–1^; 307.5 mAh g^–1^ at 20 A g^–1^	254.8 mAh g^–1^ after 2000 cycles at 5 A g^–1^		14	[57]
Bi_2_Te_3_@rGO@NC	322.7 mAh g^–1^ at 50 mA g^–1^; 37.8 mAh g^–1^ at 2 A g^–1^	107.7 mAh g^–1^ after 100 cycles at 0.2 A g^–1^		–	[67]
Bi_2_Te_3− x_ @NPCNFs	402.2 mAh g^–1^ at 50 mA g^–1^; 109.5 mAh g^–1^ at 2 A g^–1^	79.4 mAh g^–1^ after 3500 cycles at 1A g^–1^		–	[68]

#### Bismuth Sulfide and Its Composites

Bismuth sulfide (Bi_2_S_3_) is a typical layered semiconductor material with a narrow band gap (1.3–1.7 eV), which is widely used in phototubes, thermoelectric devices, photoelectric components and infrared spectrometers. Notably, the layered structure of Bi_2_S_3_ ensures the rapid insertion of K^+^. Moreover, Bi_2_S_3_ has a high theoretical capacity (625 mAh g^−1^), which makes it a potential anode material for PIBs.

**Fig. 6:**
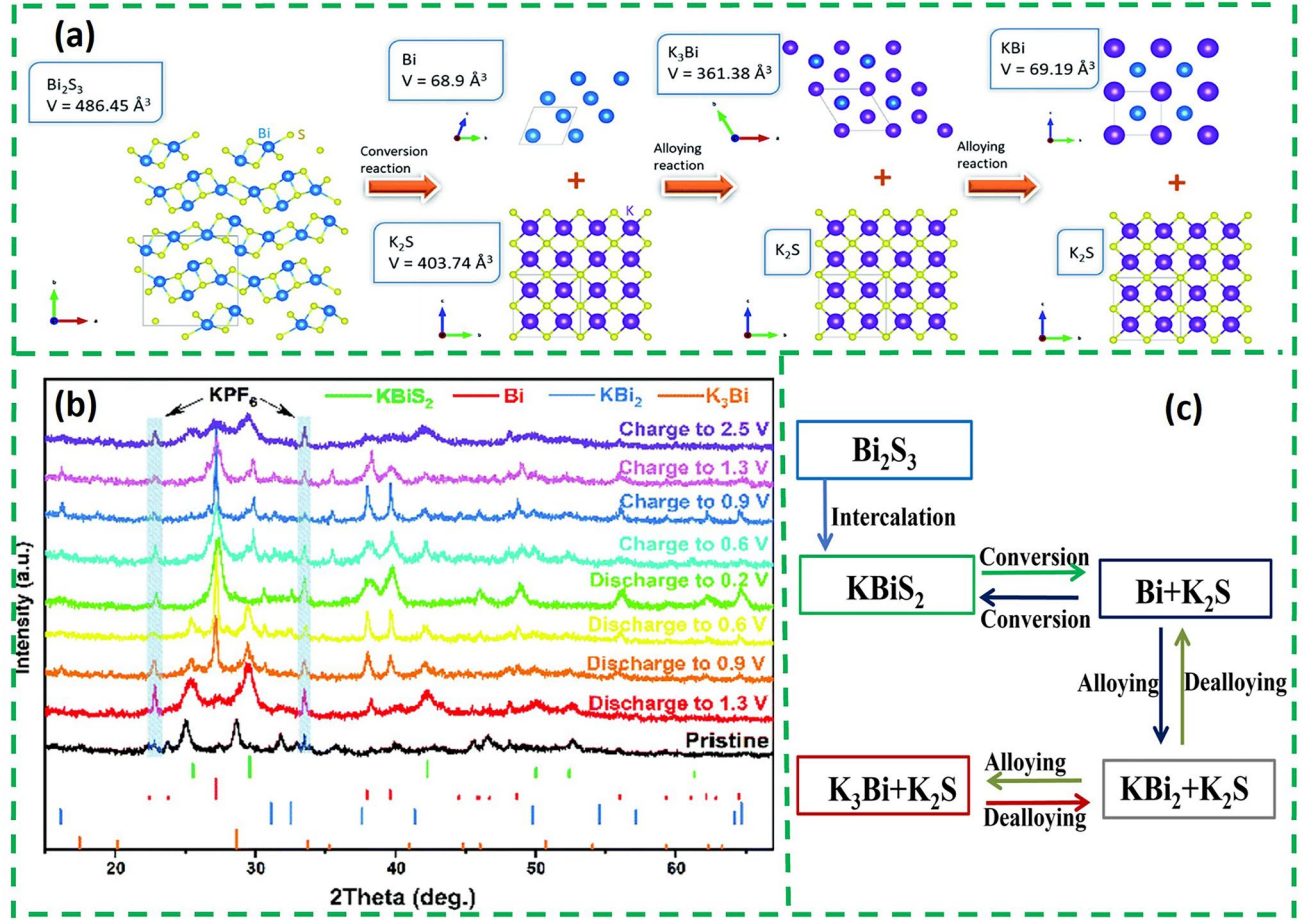
**a** Electrochemical potassium storage mechanism of Bi_2_S_3_ in the composite electrode and volume changes; the insets show the detailed crystalline parameters computed by DFT [59]. Copyright 2020, Royal Society of Chemistry. **b** Ex situ XRD patterns of the Bi_2_S_3_@RGO [60]. Copyright 2024, Clearance Center, Inc. **c** Schematic illustration of K^+^. storage mechanism of Bi_2_S_3_

Based on the energy storage mechanism of Bi_2_S_3_ in LIB and SIB, a work inferred the potassium storage mechanism of Bi_2_S_3_ by density functional theory (DFT) calculations (Fig. 6a) [59]. The DFT calculations show that in the first stage, Bi_2_S_3_ forms a K_2_S phase with a unit cell volume of 403.74 Å^3^. Then further potassiumization results in the formation of K_3_Bi with a unit cell volume of 361.38 Å^3^ from the metal Bi, followed by the formation of the KBi phase in the final stage.

However, different from this, the potassium storage mechanism of Bi_2_S_3_@RGO composite material was revealed by ex situ XRD and HRTEM [60]. As shown in the XRD (Fig. 6b), the diffraction peak of KBiS_2_ can be observed at 1.3 V. Then the KBiS_2_ peak weakens as the discharge progresses, and the Bi phase appears at 0.9 V. Finally, the KBi_2_ and K_3_Bi phases can be observed at the end of the discharge. The charging process is the reverse of the discharge process. However, the KBiS_2_ phase can be detected when fully charged to 2.5 V. This indicates that the conversion of KBiS_2_ to Bi_2_S_3_ is not completely reversible. The corresponding intercalation-conversion-alloy storage schematic is shown in Fig. 6c. The difference in the above two works lies in the presence of irreversible reactions as well as different intermediates, which may be related to electrolytes and characterization.

The anode of Bi_2_S_3_ has the disadvantages of enormous volume expansion, sluggish K^+^ storage kinetics, “shuttle effect” of polysulfide and the dissolution of intermediate products. To address these challenges, strategies such as nanostructure engineering, and rational structural designs (including composite formation and surface coating) are applied to design highly efficient and advanced Bi_2_S_3_ electrodes.

**Fig. 7:**
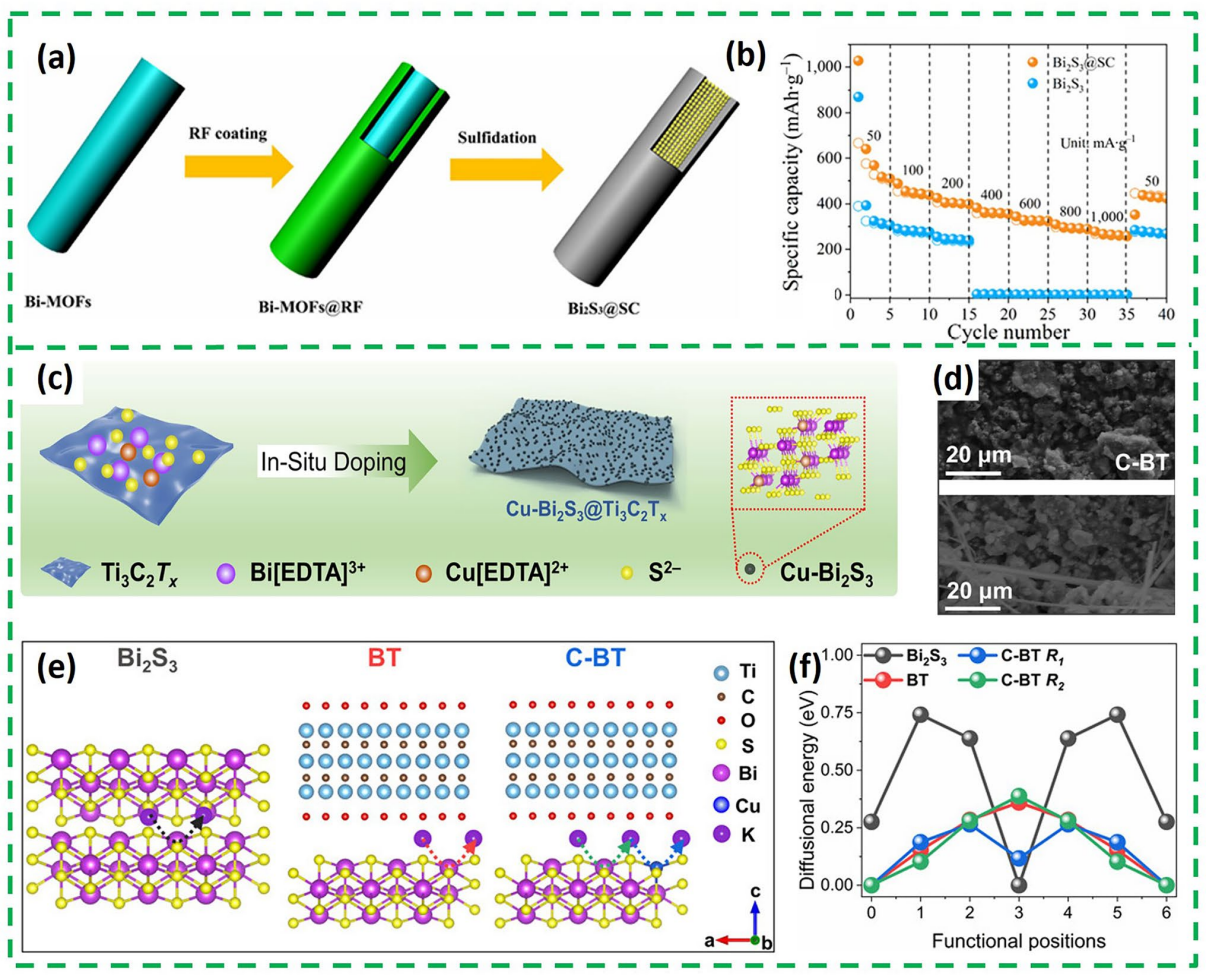
**a** Schematic illustration for the synthesis process of the core–shell-structured Bi_2_S_3_@SC. **b** Rate capability [61]. Copyright 2021, Tsinghua University Press and Springer-Verlag GmbH, Germany. **c** Schematic illustration of the synthesis of CBT. **d** SEM images of C-BT fresh and cycled electrodes after 200 cycles at 0.5 A g^−1^. **e** K^+^ diffusion routes and **f** corresponding K^+^ diffusion energy barrier in Bi_2_S_3_, BT, and C-BT [62]. Copyright 2024, Wiley

Bi_2_S_3_ coated with sulfur-doped carbon shells (Bi_2_S_3_@SC) was prepared through the pyrolysis and sulfuration of Bi-MOF (Fig. 7a) [61]. The outer sulfur-doped carbon shell not only acts as a buffer, but also alleviates the "shuttle effect" of the polysulfide by isolating the Bi_2_S_3_ from the electrolyte. Based on these structural advantages, Bi_2_S_3_@SC obtained a higher specific capacity and rate performance (Fig. 7b). Furthermore, in addition to optimize the external structure, optimizing the internal structure through doping is also an important strategy to improve the potassium storage performance of Bi_2_S_3_. In a study, Cu^2+^-doped Bi_2_S_3_ anchored on highly conductive 2D Ti_3_C_2_T_x_ nanosheets (C-BT) was prepared by co-precipitation [62] (Fig. 7c). The interconnected Ti_3_C_2_T_x_ can build a 3D structure to prevent aggregation of Bi_2_S_3_ nanoparticles and stabilize the electrode structure by slowing down the volume change throughout the cycling process, and the structure is still free of obvious cracks and aggregation after 200 cycles of circulation at 0.5 A g^−1^ (Fig. 7d). As shown in Fig. 7e, compared with Bi_2_S_3_ and BT, C-BT provided an additional diffusion route in the doping site (C-BT R_1_). The diffusional energies of these routes decrease according to Bi_2_S_3_, C-BT R_2_, BT and C-BT R_1_, and the R_2_ (0.39 eV) of C-BT is slightly higher than that of BT (Fig. 7f). All this suggests that the recombination of Ti_3_C_2_T_x_ can enhance the K^+^ transport in the intercalation stage and doping Cu^2+^ can act as the active site to selectively promote K^+^ insertion without affecting the diffusion of surrounding K^+^.

#### ***Bismuth Selenide (Bi***_***x***_***Se***_***y***_***) and Its Composites***

Bismuth selenides (Bi_x_Se_y_), such as BiSe, Bi_2_Se_3_, Bi_4_Se_3_ and Bi_3_Se_4_, are considered excellent candidates for electrical applications due to their layered crystal structure, high reversible capacity and relatively weak shuttle effect.

**Fig. 8:**
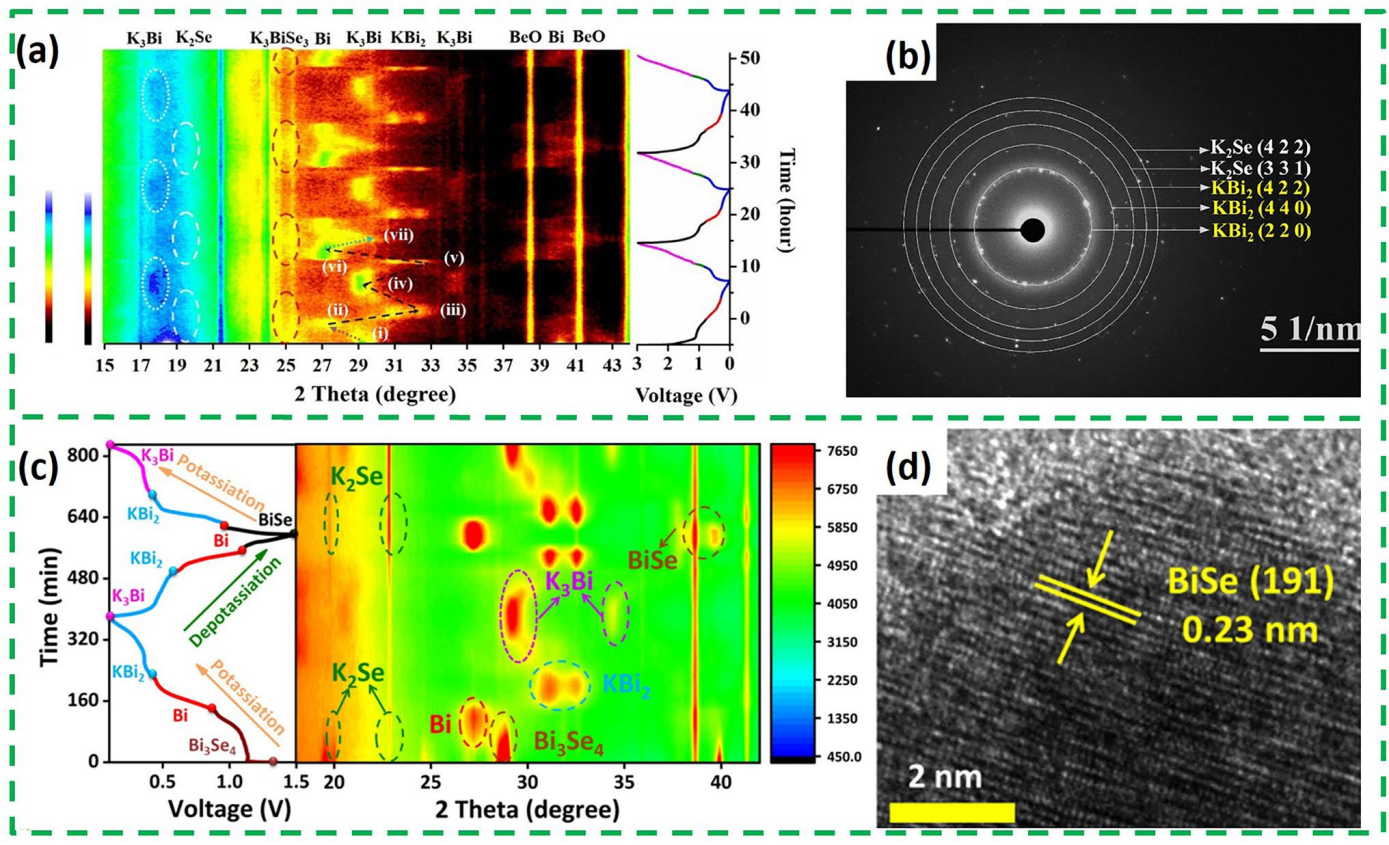
**a** In situ contour plot of the operando XRD result of Bi_2_Se_3_@NC@rGO electrode during the K^+^ insertion/extraction process of the initial three cycles. **b** Selected area electron diffraction pattern when discharging to 0.6 V during the first cycle [63]. Copyright 2021, Elsevier B.V. **c** In situ XRD patterns of Bi/Bi_3_Se_4_@CNR electrode. **d** Ex situ HRTEM of BiSe [57]. Copyright 2023, Science Press

Similar to Bi_2_O_3_, Bi_x_Se_y_ is also based on a conversion-alloying dual mechanism to storage potassium. The in situ XRD results of Bi_2_Se_3_ are shown in Fig. 8a, where diffraction peaks of K_3_BiSe_3_, Bi, KBi_2_ and K_3_Bi were sequentially observed during the discharge process, indicating that Bi_2_Se_3_ first undergoes two consecutive transformation reactions, and then Bi and K^+^ undergo a two-step alloying reaction to form KBi_2_ and K_3_Bi. The corresponding KBi_2_ phase can also be detected by selected-region electron diffraction (Fig. 8b). The charging process is the inverse of the discharging process, and the structure of Bi_2_Se_3_ reappears after the end of charging [63]. Bi_4_Se_3_ is the same as Bi_2_Se_3_, with a highly reversible dual potassium storage mechanism based on the transformation/alloying reaction [64]. However, the storage mechanism of Bi_3_Se_4_ differs from the above two substances. Recently, the potassium storage mechanism of Bi_3_Se_4_ was revealed by in situ XRD and HRTEM [57]. As shown in Fig. 8c, the initial discharge process is the same as Bi_2_Se_3_ and Bi_4_Se_3_. However, Bi_3_Se_4_ was not regenerated during the initial charging process, and a new peak of BiSe was detected, which can also be observed in TEM (Fig. 8d). This suggests that Bi_3_Se_4_ undergoes an irreversible conversion reaction during potassium storage. It is noteworthy that K_2_Se phase was always present throughout the charging/discharging process, which also suggests that the transformation of Se is irreversible. In summary, the difference between the three types of Bi_x_Se_y_ mentioned above is the reversibility of the transformation reaction. For example, Bi_2_Se_3_ produces the intermediate K_3_BiSe_3_, and the storage process of potassium is highly reversible. However, the reversal reaction of Bi_3_Se_4_ is irreversible.

**Fig. 9:**
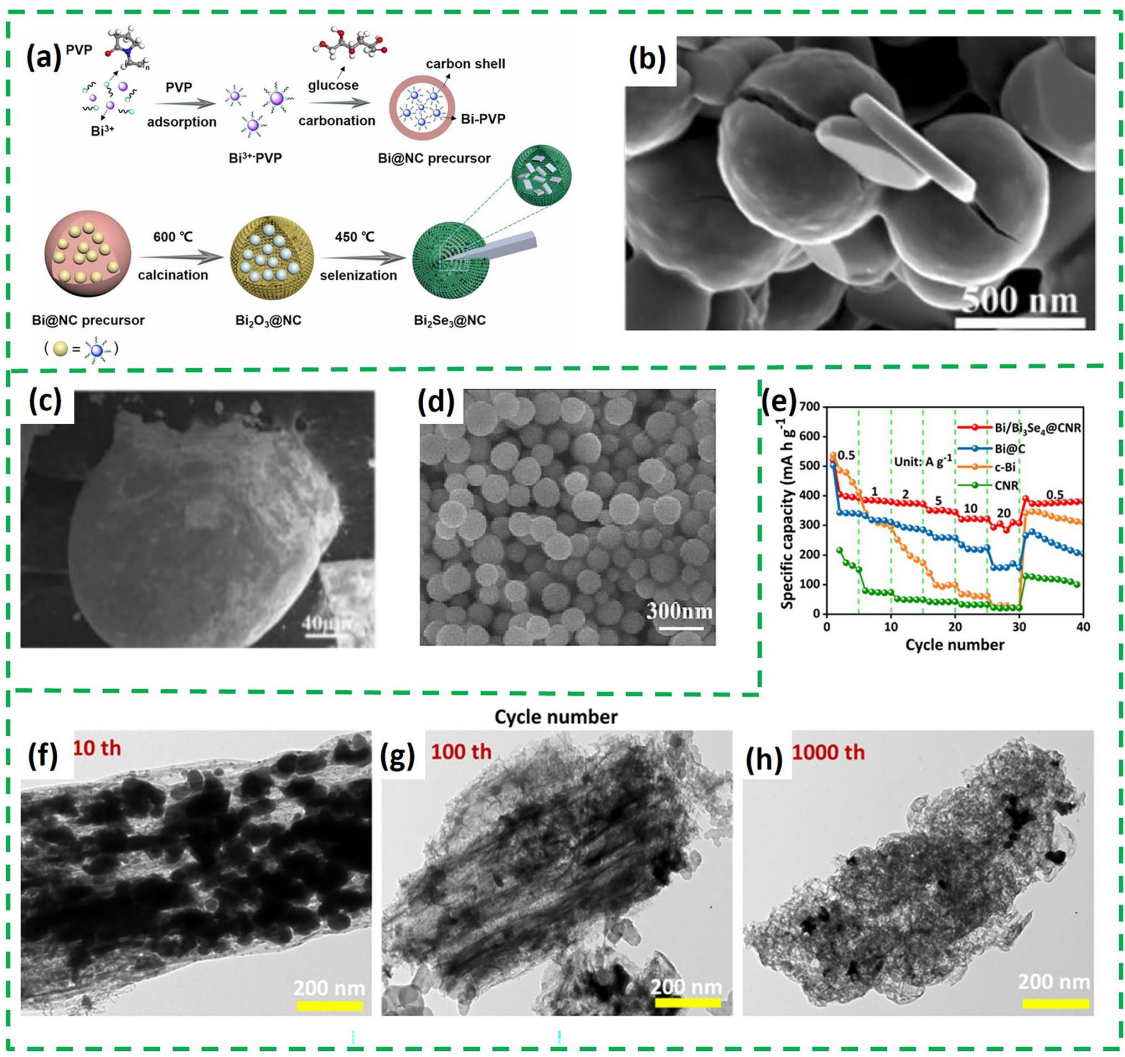
**a** Schematic illustration for the synthesis processes of Bi_2_Se_3_@NC. **b** TEM images of Bi_2_Se_3_@NC [65]. Copyright 2022, Elsevier Ltd. SEM of bismuth **c** in the Bi@C sample and **d** in Bi@Bi_4_Se_3_@C [64]. Copyright 2023, Elsevier B.V. **e** Rate performances. **f–h** TEM of Bi/Bi_3_Se_4_@CNR after 10 cycles, 100 cycles and 1000 cycles at 5 A g^−1^ [57]. Copyright 2023, Science Press

Bi_x_Se_y_, which provides high capacity, also causes severe volume expansion and electrode crushing due to volume changes during potassium storage as well, which limits its application in PIBs. Many Bi_x_Se_y_ composites have been reported for improving the performance of PIBs. Compositing Bi_x_Se_y_ with carbonaceous materials is an effective strategy. A study synthesized Bi_2_Se_3_@NC composite materials by simple solvothermal and annealing methods (Fig. 9a) [65]. During the annealing process, Bi_2_Se_3_ nanosheets grow outward and eventually form carbon shell lollipop-like composites (Fig. 9b). The lollipop-like Bi_2_Se_3_@NC has a large specific surface area, which effectively improves the kinetics. In addition, Bi_2_Se_3_ is in situ encapsulated in a carbon shell composite material, which can accommodate and limit volume expansion to a large extent. Finally, Bi_2_Se_3_@NC obtained a specific capacity of 167 mAh g^−1^ after 2000 cycles at 500 mA g^−1^.

In addition, Bi_x_Se_y_ is more attractive to Bi than carbon materials, so it can be used as a protective layer to achieve uniform and nanosized Bi particle growth. A group prepared Bi nanospheres protected by Bi_4_Se_3_ and a carbon layer (Bi@Bi_4_Se_3_@C) by in situ selenization of Bi nanospheres [64]. As shown in Fig. 9c, d, Bi grows smaller and more uniform with the existence of Bi_4_Se_3_. The active Bi_4_Se_3_ coating not only solved the problem of excessive growth of low melting point Bi metal negative electrode, but also boots the K^+^ diffusion kinetics and alleviates the volume expansion during electrode cycling. Moreover, as a potassium storage active substance, the Bi_4_Se_3_ coating layer improves the specific capacity of the electrode. Furthermore, a rational structural design can take K_2_Se, a conversion product of Bi_x_Se_y_, as a buffer substance to effectively improve the stability of the PIBs. Bi/Bi_3_Se_4_ nanoparticles distributed in carbon nanorods (Bi/Bi_3_Se_4_@CNR) also prepared by pyrolysis and selenization of Bi-MOF [57], in which Bi_3_Se_4_ nanoparticles with high electrical conductivity and high activity also provided high specific capacity during potassium storage (Fig. 9e). Besides, the synergistic effect of K_2_Se and the buffer space inside the carbon rod can maintain the structural stability of Bi/Bi_3_Se_4_@CNR during repeated potassium-ion intercalation/de-intercalation. As shown in Fig. 9f-h, the initial structure is retained even after 1000 cycles at 5 A g^−1^.

#### ***Bismuth Tellurides (Bi***_***x***_***Te***_***y***_***) and Its Composites***

The theoretical capacity of layered Bi_2_Te_3_ is 402 mAh g^−1^. And compared to sulfides and selenides, on the one hand, layered Bi_x_Te_y_ has a greater density, endowing them with higher bulk energy density; on the other hand, Bi_x_Te_y_ has a higher electrical conductivity, which gives them faster diffusion kinetics of K^+^ [66]. In summary, in the field of potassium storage, Bi_x_Te_y_ is currently less reported, but Bi_2_Te_3_ has great potential.

**Fig. 10:**
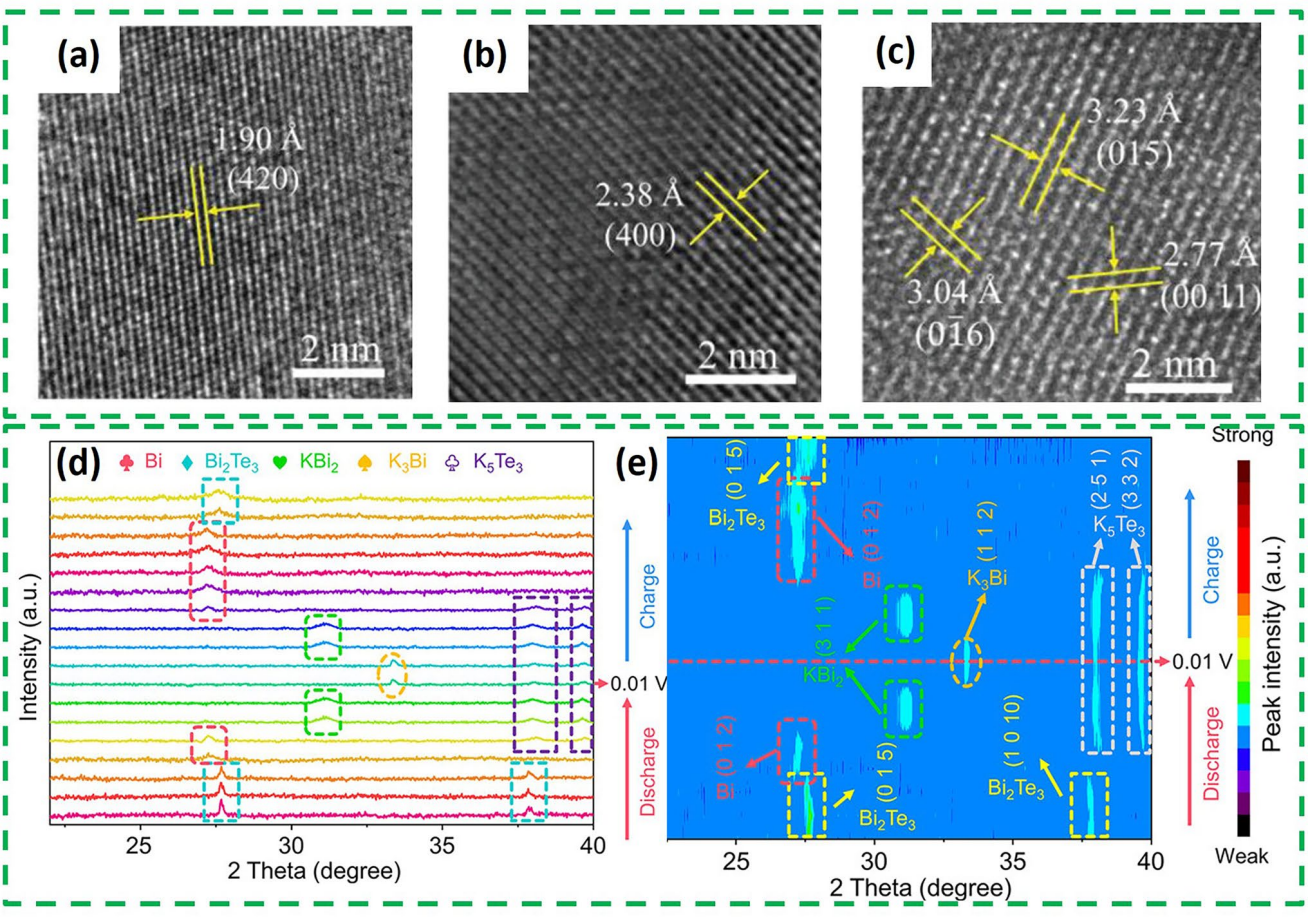
HRTEM image **a** after initial fully discharging to 0.01 V, **b** after initial charging to 1.1 V, **c** after initial fully charging to 3.0 V [67]. Copyright 2023, Wiley–VCH GmbH. **d** Ex situ XRD patterns and **e** corresponding contour image of the Bi_2_Te_3–x_@NPCNFs electrode during the first cycle [68]. Copyright 2024, American Chemical Society

As for the potassium storage mechanism, ex situ TEM and high-angle annular dark field (HAADF-STEM) demonstrated that Bi_2_Te_3_ is a typical conversion-alloying dual potassium storage mechanism [67]. Lattice fringes with crystal face spacing of 1.9 Å can be observed in HRTEM (Fig. 10a) when discharge to 0.01 V, corresponding to the (4 2 0) plane of K_3_Bi phase. When charged to 1.1 V, a plane spacing of 2.38 Å was observed (Fig. 10b), which is the plane spacing belonging to the (4 0 0) plane of KBi_2_. When fully charged to 3 V, Bi_2_Te_3_ can be seen in the TEM (Fig. 10c). All of these suggest that the fully discharge product is K_3_Bi, and KBi_2_ is the intermediate product in the alloy/dealloying process. The specific storage processes include: (conversion) Bi_2_Te_3_ + 6 K^+^  + 6e^−^ = 2Bi + 3K_2_Te, (alloying) 2Bi + K^+^  + e^−^ = KBi_2_, KBi_2_ + 5 K^+^  + 5e^−^ → 2K_3_Bi. Besides, a study revealed the potassium storage mechanism of Bi_2_Te_3-x_ by ex situ XRD and TEM [68]. Different to Bi_2_Te_3_, the XRD (Fig. 10d, e) can observe that the signals of the (0 1 5) and (1 0 10) crystal planes of Bi_2_Te_3-x_ gradually weaken and shift slightly to a lower angle during the discharge process from 2.5 to 1.2 V, which is attributed to the intercalation reaction of K insertion into K_x_Bi_2_Te_3-x_. The discharge process includes Bi_2_Te_3-x_ → K_x_Bi_2_Te_3-x_ → Bi + K_5_Te_3_, Bi → KBi_2_ → K_3_Bi. The charging process is the inverse conversion of the discharge. These results indicate that Bi_2_Te_3-x_@NPCNFs follow a highly reversible “intercalation-transformation-stepwise alloying” reaction mechanism.

However, the large volume expansion (406%) from Bi to K_3_Bi is still a serious drawback of Bi_2_Te_3_ for PIBs at present. The main strategies to address this shortcoming are compositing with carbonaceous materials, constructing nanostructures and utilizing defect engineering.

Composite with carbonaceous materials is an effective strategy to buffer the volume expansion and improve the conductivity of the electrodes. A work encapsulated Bi_2_Te_3_ nanoplates in rGO and NC layers [67]. The physicochemical encapsulation effect of rGO and NC layers as well as the synergistic effect of the Bi–O–C bonding not only suppressed the lattice stresses efficiently but also lowered the diffusional resistance of K^+^ and improved the kinetics of the transport of electrons and K^+^. Recently, a study combines innovative structural and defect engineering strategies to embed ultrafine Bi_2_Te_3-x_ nanocrystals enriched with Te vacancies into N-doped porous carbon nanofibers (NPCNFs) and applying it as PIBs anode [68]. The increased vacancies not only provide sufficient buffer space for volume expansion, but also provide additional carriers to accelerate the electron/K^+^ diffusion kinetics during charging and discharging. The 3D interconnected porous fiber structure of NPCNFs effectively disperses Bi_2_Te_3-x_ while also building fast channels for rapid electron/ion transfer and stable electrode structures.

In general, Bi_2_O_3_ and Bi_a_X_b_ (*X* = S, Se, Te) are faced with the following three problems: (1) Their potassium storage mechanisms all include the transformation-alloy dual mechanism, which will inevitably produce huge volume expansion in the process of charge and discharge, leading to poor cycling stability. (2) The reversibility of the conversion reaction is also a concern for this kind of materials. (3) The low electrical conductivity of these materials, especially Bi_2_O_3_, affects the electrochemical properties. In addition, although Bi_a_X_b_ (*X* = S, Se, Te) is layered materials, there are also some differences in potassium storage performance when *X* is S, Se, Te. First, the theoretical capacity of Bi_2_S_3_ (625 mAh g^−1^), Bi_2_Se_3_ (491 mAh g^−1^), and Bi_2_Te_3_ (402 mAh g^−1^) decreased successively. Second, Bi_2_S_3_ faces a serious shuttling effect problem. Intermediates generated during the potassium storage process may dissolve into the electrolyte, which can lead to material deactivation and rapid capacity degradation. Third, compared with the other two types of materials, Bi_2_Se_3_ has a larger layer spacing, which is conducive to the embedding of K^+^. Fourth, Bi_2_Te_3_ has higher electrical conductivity than the other two types of materials. In short, all kinds of materials have advantages and disadvantages in potassium storage. Many strategies have been developed to address these issues and progress has been made.

However, in order to build more efficient and stable transition metal chalcogenides (TMCs) PIBs anodes, there are still some problems that need to be solved. Firstly, the current research on the modification of TMCs mainly focuses on the composite with carbonaceous materials, which is relatively homogeneous. More promising bismuth-based sulfur compounds should be developed from the optimization of internal and external environments, such as the construction of more complex nanostructures and composite structures to optimize their electrical properties from an atomic perspective. In addition, theoretical studies on TMCs, especially the internal potassium storage mechanism of Bi_x_Te_y_, still need to be further strengthened.

### Bismuth-Based Alloys and Their Composites

The alloying materials are one of the ideal anodes for alkali metal ion batteries due to high theoretical capacity and low operating potential. The constituent elements of alloy anode materials mainly locate in Groups IV A and V A [75]. The synergy effects between different elements lead to more stable structures of materials. Bismuth-based alloys combine the stable crystal structure of the matrix metallic Bi with the fast kinetics, contributed by reinforcement, as well as low thermal conductivity, low melting point and non-toxicity. The different elements act as buffer layers of volume expansion each other. Bismuth-based alloys have received much attention in recent years [76]. According to the available literatures, the preparation methods of bismuth-based alloys include freeze-drying and pyrolysis method, solvothermal approach, laser irradiation technique, ball milling method, hydrolysis method, sputtering method and chemical dealloying method. Although bismuth-based ternary alloys have been studied in rechargeable batteries for a long time, bismuth-based binary alloys are more commonly reported in PIBs than ternary alloys and they are mostly prepared by dealloying from ternary alloy precursors [77]. During the alloying process, Bi_x_M_y_ (M is the reinforcer) alloy anodes in PIBs generally undergoes the reaction of K and (Bi, M) to generate K(Bi, M) at first, with K embedding into (Bi, M) lattice; then, K(Bi, M) completely transforms into K_3_(Bi, M). Steps are the opposite for the dealloying process [78].

**Fig. 11:**
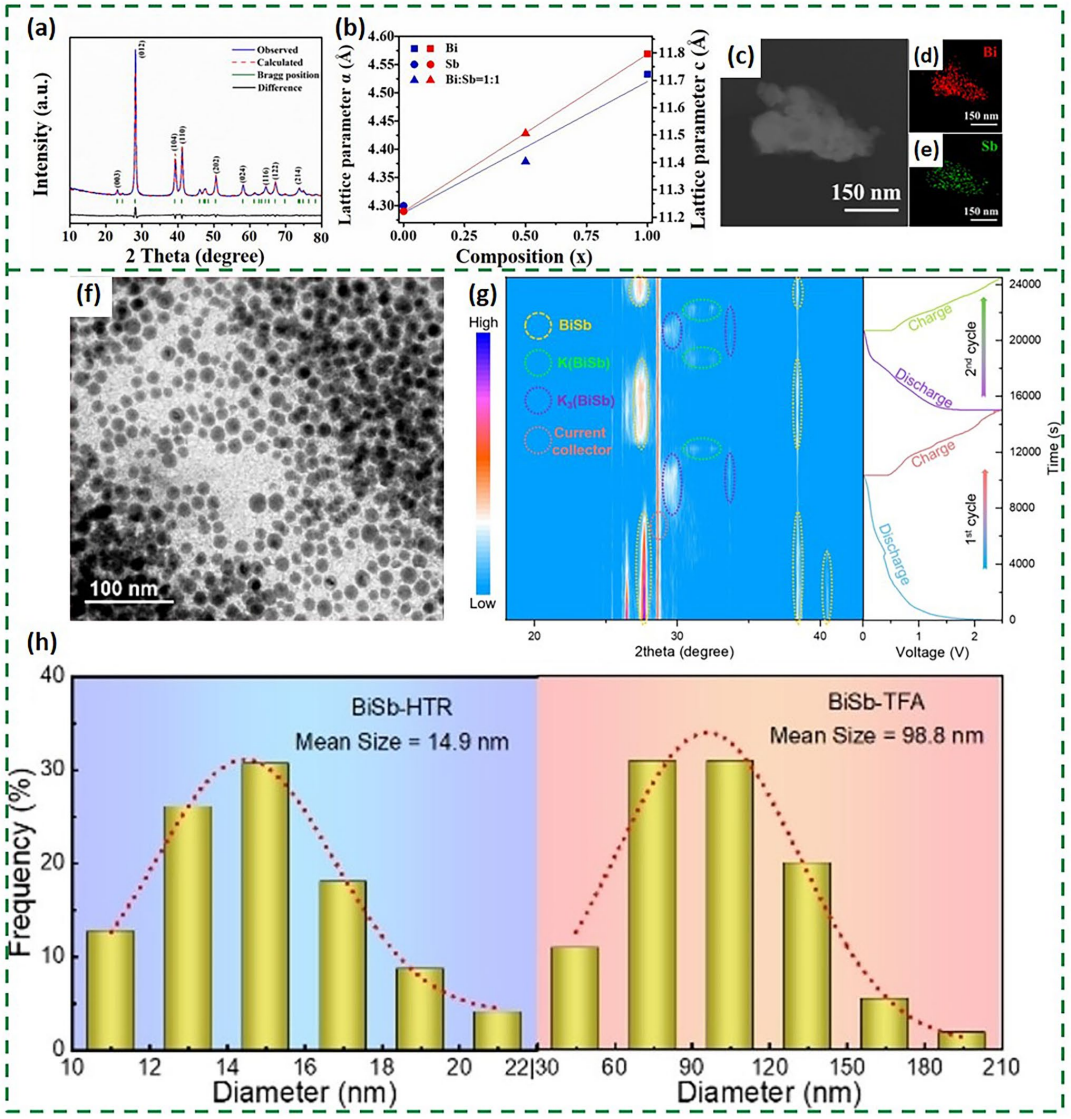
**a** Rietveld refinement of the XRD pattern. **b** Dependence of the lattice parameters *a* (blue) and *c* (red) on the *x* of the BiSb. **c–e** EDS mapping images of Bi and Sb [79]. Copyright 2021, Elsevier. **f** TEM image of BiSb synthesized by HTR (BiSb-HTR). **g** Contour plot of the in situ XRD patterns of BiSb-HTR anode and the corresponding charge/discharge curve during the 1st and 2nd K^+^ insertion/extraction cycles. **h** Nanoparticle size distributions of BiSb-HTR and BiSb-TFA derived from the TEM images [80]. Copyright 2023, John Wiley and Sons

It is common for metallic Bi to form alloy anodes with Sb, Sn and Cu through metallic bonds for achieving high theoretical specific capacity. A series of alloy materials with different melting points and properties can be obtained in modulating the percentage of these metals. When they serve as anodes in PIBs, BiSb alloy exhibits decent potassium storage capacity due to the following points. On the one hand, Bi and Sb are in the same main group and share equal outermost electrons and therefore have similar physical chemical properties. They can form solid solutions according to any molar ratio, allowing the melting point and the capacity of alloy, etc. to be controlled by adjusting the proportion of components. On the other hand, the relative atomic mass of Sb is smaller than that of Bi, which can improve the specific capacity based on sole metallic Bi. Previously, it was reported that BiSb nanocrystals were obtained by ball milling of Bi and Sb powders on a ratio of 1:1 and fast charging and discharging were realized as anodes for PIBs [79]. Due to the larger Bi atoms replacing the smaller Sb atoms, the diffraction peaks of the submicron BiSb alloys were lower than the standard peaks of Sb powders (Fig. 11a). The energy-dispersive spectroscopy (EDS) mapping illustrated in Fig. 11c-e shows a uniform distribution of Bi and Sb elements with a 1:1 atomic ratio, which was consistent with the calculated lattice parameters (Fig. 11b). The robust BiSb nanocrystalline aggregates have interconnected conductive grains, providing ideas for the future design of alloy-type anodes.

**Fig. 12:**
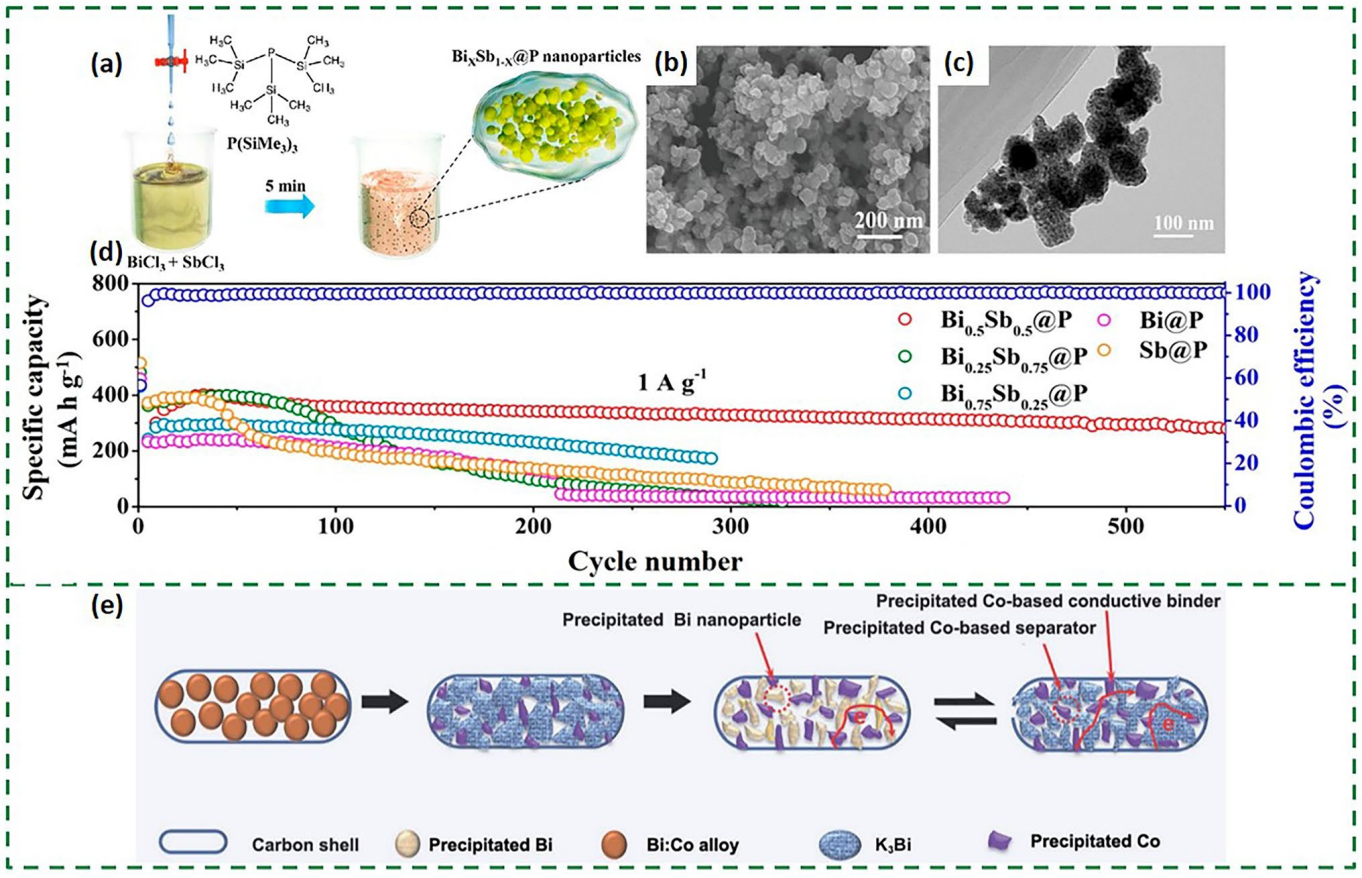
**a** Schematic illustration of Bi_x_Sb_1-x_@P synthesis. **b**, **c** SEM and TEM images of the Bi_0.5_Sb_0.5_@P nanocomposites. **d** Cycling performance of Bi_*x*_Sb_1–*x*_@P electrodes at rates of 1 A g^−1^ [81]. Copyright 2020, ACS Publications. **e** Schematic diagram illustrating the phase and morphology changes of the metastable Bi:Co@C structures during the discharge/charge processes [84]. Copyright 2022, Tsinghua University Press

However, alloy-based anodes generally suffer from drastic volume expansion, causing the electrode to deform, chalk and collapse. In addition, alloy-based anodes are prone to produce unstable SEI films, which severely deplete potassium ion and electrolyte, inhibiting metallic ions transport and leading to rapid capacity decay. Moreover, similar to most alloy-based anodes, bismuth-based alloys are far less conductive than anode materials such as graphite, etc., which is not conducive to rapid electron transport. In order to overcome these shortcomings, various strategies such as the introduction of hollow porous nanostructure and the design of Bi_x_M_y_/C composite materials are normally employed to mitigate the volume expansion of bismuth-based alloys and receive better conductivity. The ultra-small BiSb alloy nanoparticles were developed for stimulating a strong synergy between Bi and Sb, which were bulk-loaded on N/O co-doped porous carbon nanosheets with a size of only 15 nm (Fig. 11f) [80]. The potassium storage mechanism elucidated through in situ XRD corroborates the highly reversible nature of BiSb (Fig. 11g). Furthermore, the researchers compared the BiSb alloys prepared using the conventional tube furnace annealing (TFA) method and the novel high-temperature radiation (HTR) method, which led to a new stage of efficient synthesis of alloy anodes (Fig. 11h). While the use of carbon materials to form bismuth-based alloy composites relieves stress and enhances stability, most of them are complex to prepare, and the quality of the products is difficult to control. Therefore, composites with other buffer materials can be considered to optimize the potassium storage performance of the nanoscale composites. BiSb nanocrystals embedded with phosphorus support (Bi_0.5_Sb_0.5_@P) have already been synthesized (Fig. 12a) by solution phase precipitation, thereby realizing a high-performance anode for PIBs [81]. The P carrier is capable of firmly anchoring Bi_0.5_Sb_0.5_ nanoparticles (Fig. 12b, c), and Bi_0.5_Sb_0.5_@P can be cycled 550 times at 1 A g^−1^ (Fig. 12d).

In addition to BiSb alloy, BiCo, BiFe, BiSn and BiCu bimetallic alloys have been found to be used as anodes for PIBs [82–84]. Two novel stable materials Bi_0.85_Co_0.15_@C and Bi_0.83_Fe_0.17_@C synthesized by annealing of Bi-MOF precursor were found to resolve the issue of structural collapse of the bismuth-based anode during cycling. The Co and Fe precipitates served as conductive binders, inhibiting the agglomeration of active material Bi nanoparticles (Fig. 12e). These findings may inform the formation of alloy particles of Bi substrates with new metals, such as Ni and In. As shown in Table 4, a large number of studies on bismuth-based alloy materials and their composites have further promoted the development of PIBs. While bismuth-based alloy materials currently play a pivotal role in enhancing the performance of PIBs, the role of them in mitigating volume expansion is still limited and the electrode collapse continues to get crushed. Therefore, it is still an urgent issue to design more bismuth-based alloy anode materials with excellent performances and high stability. Furthermore, it is needed to make unremitting efforts to develop high-performance bismuth-based ternary alloys for PIBs.

**Table 4:** Performance comparison of bismuth-based alloy

Bismuth-based alloy anode of PIBs				
Materials	Rate capability	Cycle capability	ICE (%)	Refs
Bi_0.5_Sb_0.5_@P	~ 375 mAh g^–1^ at 0.5 A g^–1^; 258.5 mAh g^–1^ at 6.5 A g^–1^	192 mAh g^–1^ after 1000 cycles at 0.5 A g^–1^	–	[81]
BiSb@Bi_2_O_3_/SbO_x_@C	257 mAh g^–1^ at 0.1 A g^–1^; 82 mAh g^–1^ at 5 A g^–1^	214 mAh g^–1^ after 500 cycles at 1 A g^–1^	34	[85]
Sb_0.5_Bi_0.5_@C	382 mAh g^–1^ at 0.05 A g^–1^; 200 mAh g^–1^ at 2 A g^–1^	226 mAh g^–1^ after 400 cycles at 0.5 A g^–1^	80.9	[86]
np-BiSb	500 mAh g^–1^ at 2 A g^–1^ (np-Bi_2_Sb_6_)	248.3 mAh g^–1^ after 400 cycles at 0.1 A g^–1^ (np-Bi_6_Sb_2_)	54.7 (np-Bi_6_Sb_2_)	[77]
BiSb@C	444.8 mAh g^–1^ at 0.05 A g^–1^; 152 mAh g^–1^ at 2 A g^–1^	320 mAh g^–1^ after 600 cycles at 0.5 A g^–1^	70.2	[87]
BiSb@TCS	316.5 mAh g^–1^ at 0.1 A g^–1^; 119.3 mAh g^–1^ at 6 A g^–1^	181 mAh g^–1^ after 5700 cycles at 2 A g^–1^	64.8	[88]
Bi_0.85_Co_0.15_@C Bi_0.83_Fe_0.17_@C	364 mAh g^–1^ at 0.2 A g^–1^; 178 mAh g^–1^ at 20 A g^–1^ 354 mAh g^–1^ at 0.2 A g^–1^; 253 mAh g^–1^ at 20 A g^–1^	218 mAh g^–1^ after 1000 cycles at 2 A g^–1^ 279 mAh g^–1^ after 975 cycles at 2 A g^–1^	– –	[84]
NP CuBi	325 mAh g^–1^ at 0.1 A g^–1^; 153 mAh g^–1^ at 1 A g^–1^	80 mAh g^–1^ after 300 cycles at 0.5 A g^–1^	44.1	[82]
BiSb BiSb/CNT	653.3 mAh g^–1^ at 0.05 A g^–1^; 62.9 mAh g^–1^ at 10 A g^–1^ –	236 mAh g^–1^ after 700 cycles at 0.5 A g^–1^ 184 mAh g^–1^ after 6000 cycles at 0.5 A g^–1^	72.6 –	[79]
np-InBi	–	248.6 mAh g^–1^ after 100 cycles at 0.2 A g^–1^	65.3	[89]

### Bismuth-Based Bimetallic Oxides and Their Composites

Metallic bismuth is able to form bimetal salts with transitional metals (TM), which have been widely used in anodes for LIBs and SIBs. There are also a few applications reported in papers relating to PIBs. The most common are bismuth tungstate (Bi_2_WO_6_), bismuth titanate (Bi_4_Ti_3_O_12_), bismuth vanadate (BiVO_4_) and bismuth molybdate (Bi_2_MoO_6_). The potassium storage mechanism of this type of anodes has both conversion and alloying reaction. The irreversible conversion reaction has occurred at first. In the conversion reaction, bismuth-based bimetal oxide is transformed into Bi micron-size atomic clusters, accompanied by the formation of amorphous K–TM–O matrixes and SEI films containing potassium salts and oxides. During the discharge process, Bi undergoes reversible alloying process to KBi and K_3_Bi, with the oxidation and reduction reaction of TMO to K_2_O for transitional metals [90]. BiTMO anodes are typically synthesized by direct hydrothermal method, acid etching or precipitation and can be designed as 1D nanorods, 2D nanosheets or 3D nanospheres with a variety of morphologies. Since both conversion and alloying reactions contribute to the potassium storage capacity, bismuth-based bimetal oxide anodes exhibit superior specific capacity. The synergistic effects between two metals decrease the activation energy of potassium-ion storage procedure and therefore achieve fast kinetics and effectively alleviate volume expansion and stress concentration caused by the metallic oxides [91].

**Fig. 13:**
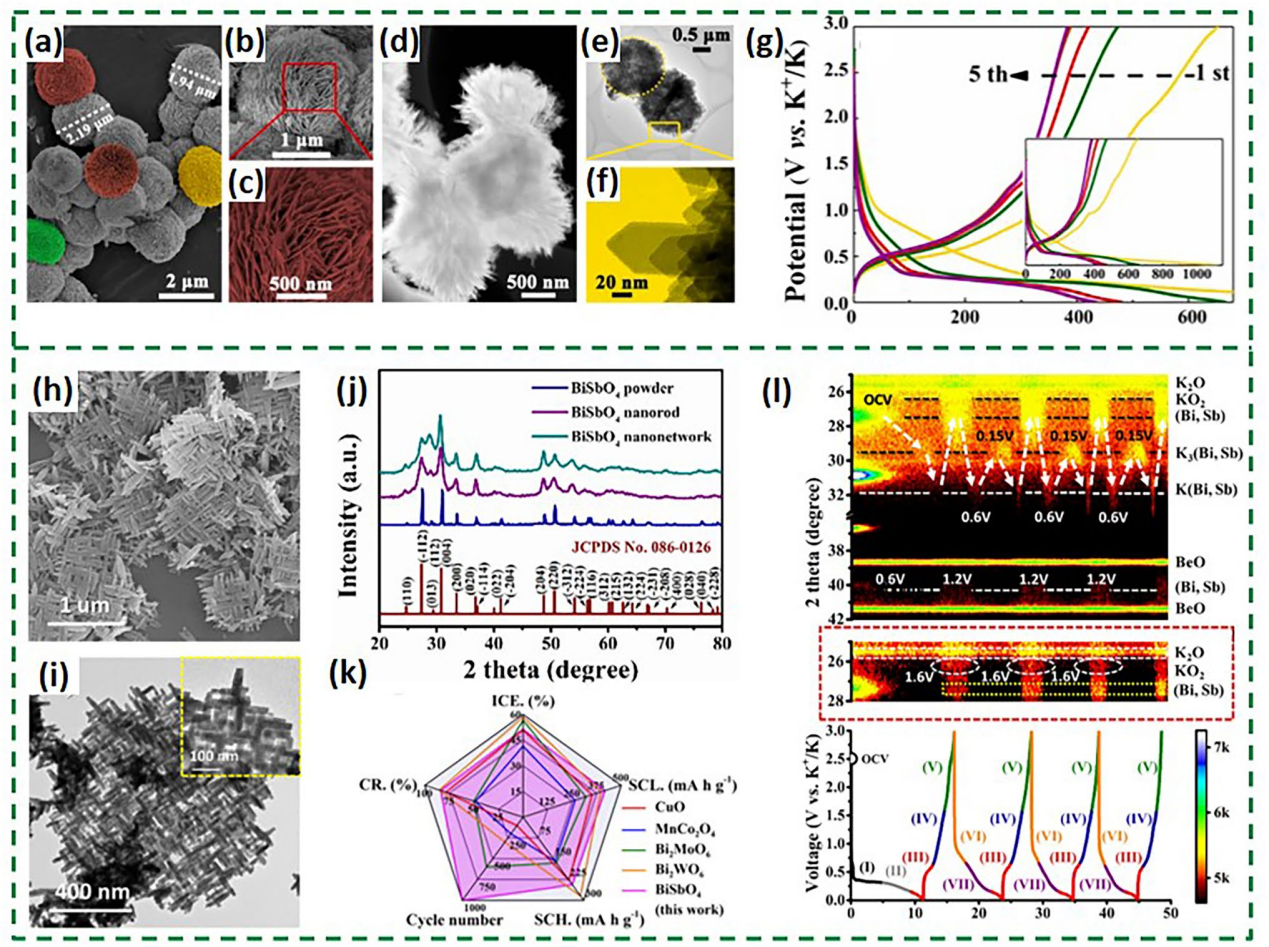
**a-f** SEM and TEM images of the BWO-MF. **g** Discharge/charge voltage profiles at 100 mA g^−1^ [92]. Copyright 2021, Published by Elsevier Ltd. **h**, **i** SEM and TEM images of BiSbO_4_ samples. **j** XRD patterns of BiSbO_4_ powders, BiSbO_4_ nanorods and BiSbO_4_ networks. **k** Comparison results of ICE, specific capacity at low and high rates (SCL and SCH), long-term cycle number and capacity retention (CR) with other previously reported anode materials of metal oxide. **l** Contour plot of the operando XRD pattern with magnified display ranged from 25° to 28° [93]. Copyright 2022, American Chemical Society

In order to develop electrode materials with desirable potassium storage performance, a large number of fruitful explorations have been carried out on metallic bismuthate. The compact Bi_2_WO_6_ micro-flowers anode material for PIBs was reported to be developed through hydrothermal method [92]. Each Bi_2_WO_6_ micro-flowers spherical particle is composed of multiple interlaced nanosheets, creating compact structures with large specific surface areas and numerous pores as shown in Fig. 13a–f, which offer high numbers of potassium-ion storage active sites. The highly active Bi nanoparticles and nanoflower skeleton obtained from Bi_2_WO_6_ conversion ensured a high reversible capacity. However, as shown in Fig. 13g, its low ICE compared to mainstream bismuth-based anode materials may be related to the severe side reactions caused by the high specific surface area, which represents a challenge faced by conversion-alloy-based materials in their early stages of development. Moreover, conventional single-metal oxides typically exhibit suboptimal charge transfer and ion diffusion kinetics. To address this challenge, the BiSbO_4_ nanonetworks comprising single crystals have been developed, which are vertically crossed, as shown in Fig. 13h, i [93]. XRD analysis of the BiSbO_4_ sample revealed the presence of diffraction peaks corresponding to the crystalline phases (Fig. 13j). As a result of nanostructural engineering that is capable of buffering volume expansion and inhibiting metal aggregation, BiSbO_4_ displays a well-balanced potassium storage capacity (Fig. 13k). In situ XRD studies elucidated the potassium storage mechanisms occurring within the BiSbO_4_ anode during the cycling process, which is the representative mechanism in bismuth-based bimetallic oxides. These studies demonstrated that the anode undergoes conversion and alloying reactions during cycling, exhibiting a good reversibility (Fig. 13l). The detailed reaction process can be summarized in the following chemical equations (Eqs. (4–6)):

4 $${\text{BiSbO}}_{4} \, + {\text{ 8K}}^{ + } + {\text{ 8e}}^{ - } \to \, \left( {{\text{Bi}},{\text{ Sb}}} \right) \, + {\text{ 4K}}_{{2}} {\text{O}}$$

5 $$\left( {{\text{Bi}},{\text{ Sb}}} \right)_{{}} + {\text{ K}}^{ + } + {\text{ e}}^{ - } \to {\text{ K}}\left( {{\text{Bi}},{\text{ Sb}}} \right)$$

6 $${\text{K}}\left( {{\text{Bi}},{\text{ Sb}}} \right)_{{}} + {\text{ 2K}}^{ + } + {\text{ 2e}}^{ - } \to {\text{ K}}_{{3}} \left( {{\text{Bi}},{\text{ Sb}}} \right)$$

**Fig. 14:**
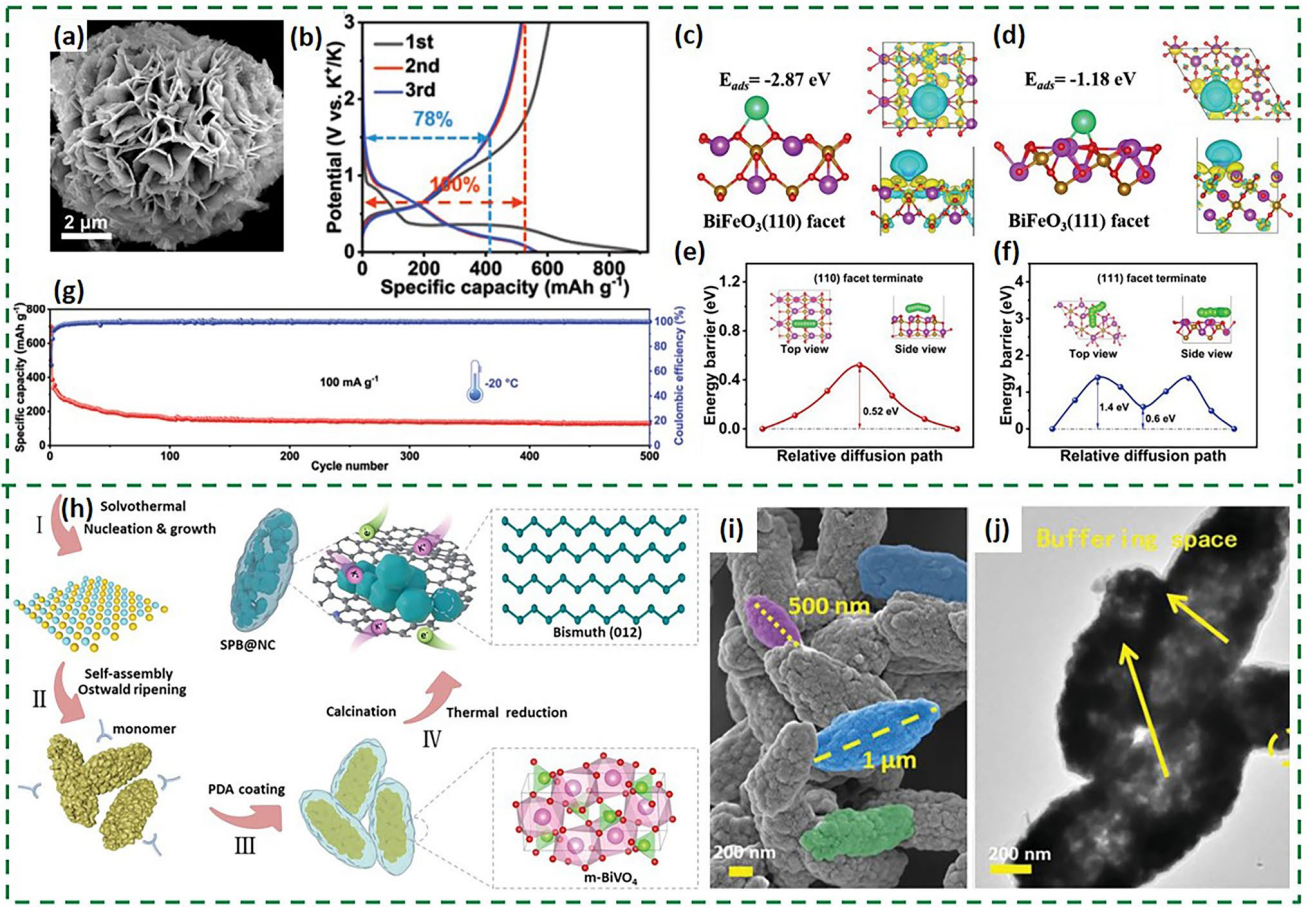
**a** SEM image of BFO-MF. **b** Galvanostatic charge/discharge profiles of BFO-MF anode at 100 mA g^−1^. **c–f** Theoretical calculation of K^+^ adsorption and diffusion on facets of BiFeO_3_. **g** Cycling performance of BFO-MF anode at − 20 °C [94]. Copyright 2024, Wiley–VCH GmbH. **h** Schematic synthesis process of SPB@NC. **i**, **j** SEM and TEM images of SPB@NC [45]. Copyright 2022, Wiley–VCH GmbH

The preparation of bismuth-based bimetallic oxides and their composites has been the subject of considerable research, but the methods currently in use are costly and low yielding. Furthermore, these materials often exhibit an unfavorable working voltage, as is the case with conventional bismuth molybdate (Bi_2_MoO_6_) microspheres. In recent years (Fig. 14a), the rhombic BiFeO_3_ microspheres were synthesized using a solvent-thermal and template method, resulting in a voltage platform of about 0.5 V by the GCD plots in Fig. 14b [94]. DFT simulations shown in Fig. 16c-f indicate that K^+^ is predominantly adsorbed and diffused on the (110) crystalline face of BiFeO_3_. Notably, the diffusion barrier of K^+^ on the (110) face at − 20 °C is nearly identical to that at 25 °C, aligning with the prolonged cycling lifespan of BFO-MF at −20 °C (Fig. 14g). The reliable operation of BFO-MF across an extensive temperature range of − 20 to 50 °C offers advanced anodes for PIBs. In addition to serving as the anode active material, BiTMO is also used for the preparation of other bismuth-based materials as the precursor. Bismuth-based anode can take on a variety of morphologies converted from BiTMO and show special structure of Bi nanoparticles. The Bi@*N*-doped carbon composite (SPB@NC) was synthesized by in situ thermal reduction of monoclinic BiVO_4_ (Fig. 14h) [45]. The fusiform BiVO_4_ precursor with multilevel structure was fabricated through self-assembly process, which was then coated with polydopamine and calcined at a high temperature. As a result, the target product SPB@NC maintained a spindle-like morphology and was internally derived from the hollow and hierarchically interconnected Bi nanoparticles (Fig. 14i, j). BiVO_4_ derivatization can ensure the robust structure and high electrical conductivity of SPB@NC, which is beneficial to the electrode stability and fast kinetics, for improving the electrochemical performances.

We deliberately collected all reported bismuth-based bimetal oxide anodes applied to PIBs, as shown in Table 5. From the above research, although bismuth-based bimetal oxide anodes are effective in reducing capacity decay and boosting cycling performance of PIBs, the depotassification of Bi will be restricted with insulation matrix K–TM–O increasing during the conversion process, resulting in declining cycling reversibility. Moreover, even nanosize bimetallic oxides are susceptible to agglomerate and damage structures, being harmful to fast K^+^ diffusion [95]. Therefore, it is significant to construct special heterogeneous structures to accelerate K^+^ transport kinetics on that basis and add carbon materials for enhancing conductivity. On the side, the selection of well-matched electrolytes to mitigate SEI films and heteroatom doping is also prospective directions for future reference, which may further optimize the electrochemical performance of bismuth-based bimetal oxides [96, 97]. In addition, the application potential of bismuth-based bimetallic sulfides, selenides and other derived materials with similar potassic/de-potassic reactions is worth exploring.

**Table 5:** Performance comparison of bismuth-based bimetal oxide

Bismuth-based bimetal oxide anode of PIBs				
Materials	Rate capability	Cycle capability	ICE (%)	Refs
Bi_2_MoO_6_	319.8 mAh g^–1^ at 0.05 A g^–1^; 165.3 mAh g^–1^ at 0.5 A g^–1^	121.7 mAh g^–1^ after 600 cycles at 0.1 A g^–1^	56.55	[98]
BWO-MF	700 mAh g^–1^ at 0.1 A g^–1^; 253 mAh g^–1^ at 1 A g^–1^	216 mAh g^–1^ after 300 cycles at 1 A g^–1^	58.4	[92]
BiSbO_4_	331.5 mAh g^–1^ at 0.05 A g^–1^; 167.1 mAh g^–1^ at 1 A g^–1^	256.5 mAh g^–1^ after 1000 cycles at 0.5 A g^–1^	57.4	[93]
BFO-MF	560 mAh g^–1^ at 0.05 A g^–1^; 279 mAh g^–1^ at 1 A g^–1^	230 mAh g^–1^ after 5000 cycles at 0.5 A g^–1^	67.9	[94]

### Bismuth Oxyhalide (BiOX, X = Cl, Br, I) and Their Composites

BiOX is a group of semiconductor compounds with special layered structures, in which [X–Bi–O–Bi–X] layers stack along the *C*-axis [99]. Non-toxic and corrosion-resistant BiOX materials have nano-sized effects, suitable redox potential and stable chemical properties. Due to their impressive physicochemical properties and suitable energy band structures, BiOX materials are widely used in photocatalysis [100]. On this basis, the semiconductor material BiOX is rich in oxygen vacancy through defective modification. Recently, BiOX have been increasingly researched as novel electrode materials for alkali metal ion batteries because of the fast ion transport and high theoretical capacity provided by their two-dimensional van der Waals heterostructures [101]. It is summarized in the available literatures that the popular methods for preparing BiOX materials include solvothermal (hydrothermal), hydrolysis, precipitation, sol–gel and soft template methods [102], which can produce significant differences in morphology and structure. The morphologies of reported BiOX materials contain nanosheets, nanoflowers, hollow nanostructures, etc. Different morphologies of BiOX have an important influence on the potassium storage application by changing the stability of crystal structure and heterogeneous structure. Therefore, the key to obtaining high-performance BiOX materials is to find an appropriate synthetic method. In contrast to anodes employed as LIBs and SIBs, the energy storage mechanism of BiOX is realized through initial irreversible conversion reaction (BiOX → Bi) and later reversible alloying reaction (K_x_Bi_y_) [103]. But in some cases, it is possible to happen reversible conversion of Bi to BiOX. BiOCl reappeared as the depotassiation went further in the study of the potassium storage mechanism [104]. The bismuth particles produced by the conversion reaction contain abundant defects, which are conducive to the rapid storage of K^+^ as active sites [105]. The internal covalent bonds within BiOX materials are strong, while the interlayer van der Waals forces are weak. Hence, on the one hand, a mass of interlayer voids can provide sufficient rapid K^+^ diffusion channels. On the other hand, the weak van der Waals forces lead to excellent mechanical flexibility of electrodes, to the benefit of accommodating potassium ion with large radius and maintaining the complete structures of anode materials.

**Fig. 15:**
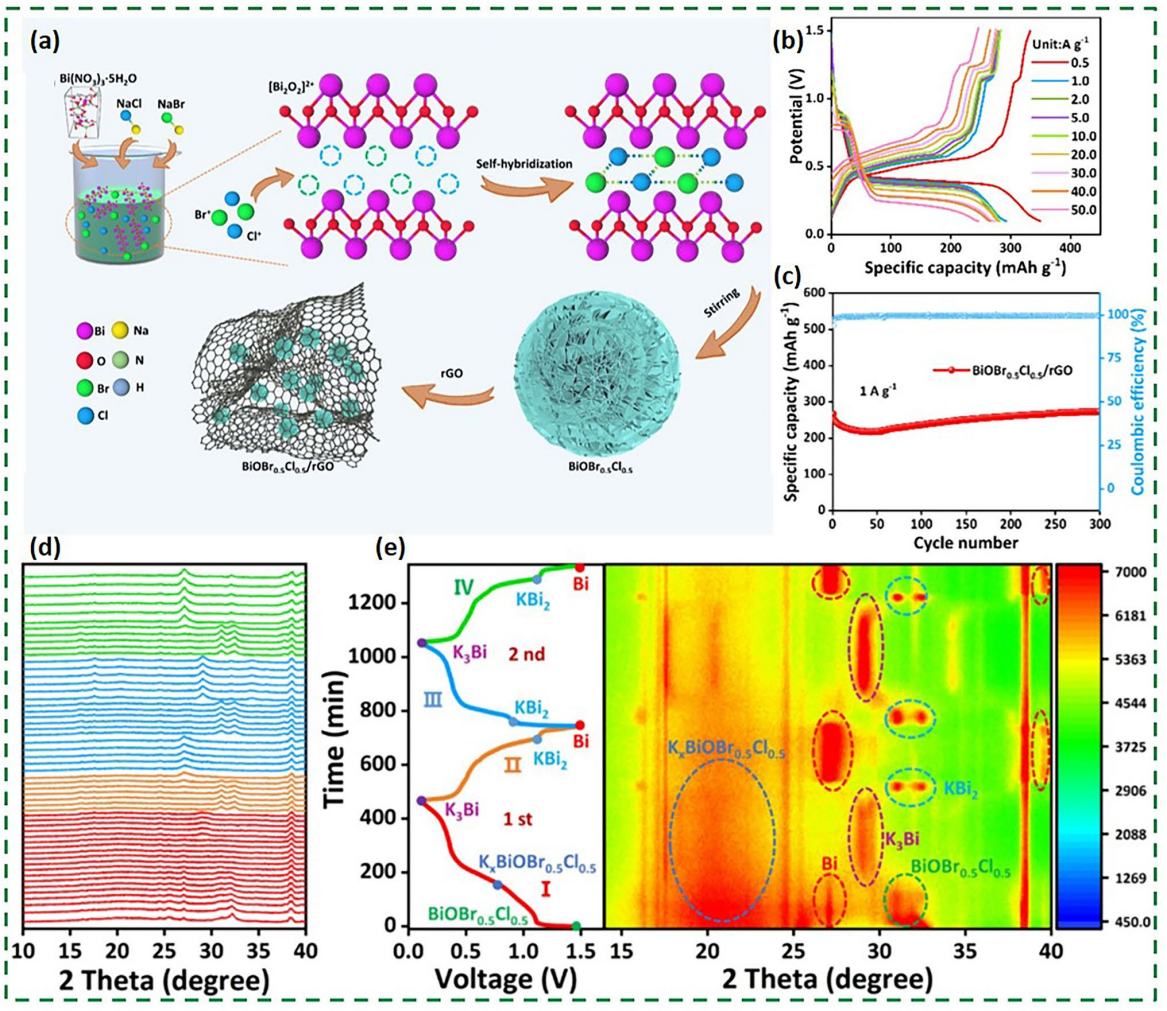
**a** Schematic synthesis process of BiOBr_0.5_Cl_0.5_/rGO. **b** GCD profiles of BiOBr_0.5_Cl_0.5_/rGO at different current densities. **c** Cycle performance at 1 A g^−1^. **d**, **e** The in situ XRD patterns of BiOBr_0.5_Cl_0.5_/rGO [106]. Copyright 2024, Elsevier Inc

**Fig. 16:**
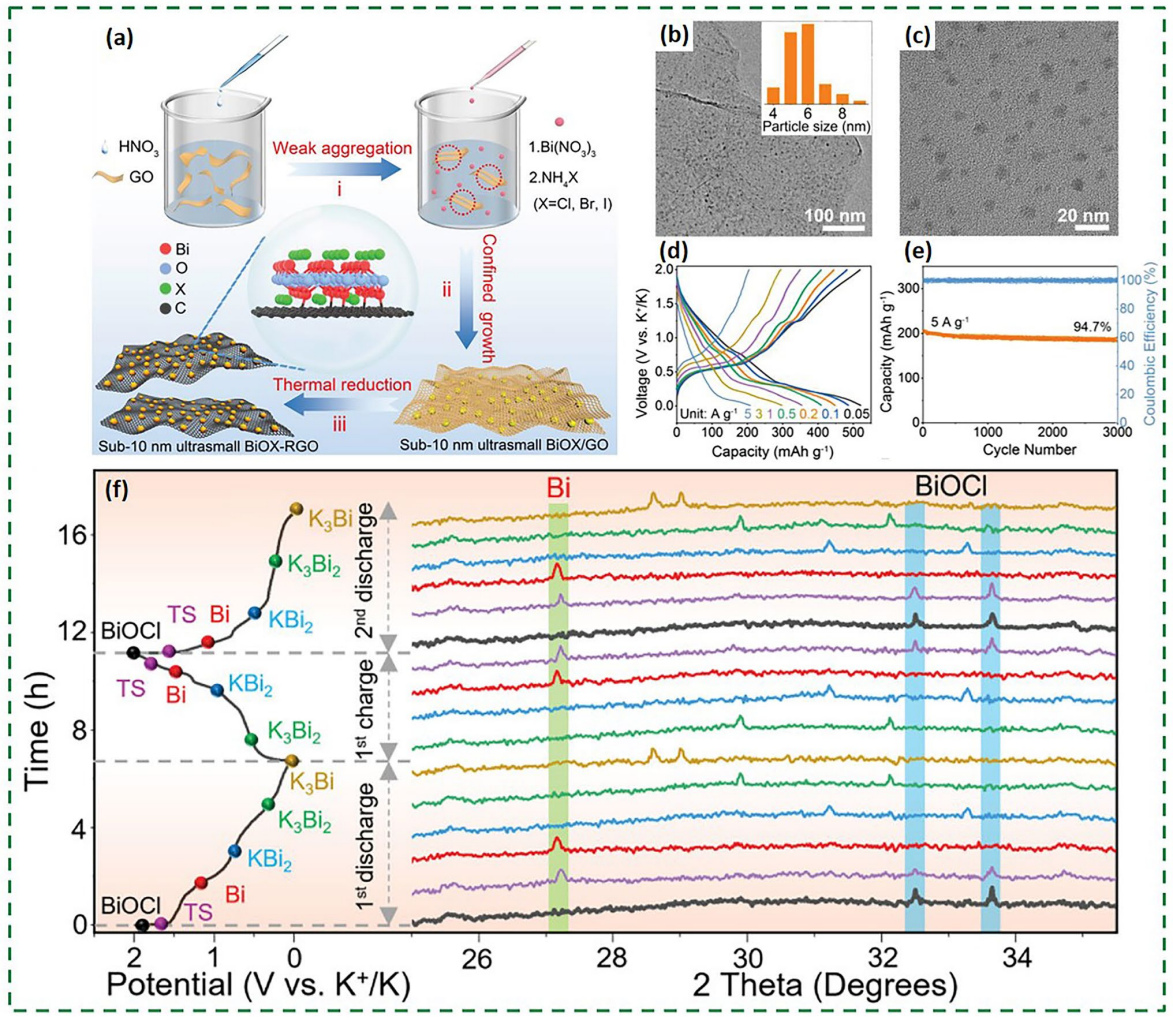
**a** Schematic of the space-confined synthesis of BiOX-RGO. **b**, **c** TEM images of S-BiOCl-RGO with the inset showing the size distribution of BiOCl nanocrystals. **d** Galvanostatic charge/discharge curves of S-BiOCl-RGO anodes at different current densities. **e** Cycling performance of S-BiOCl-RGO at 5 A g^−1^. **f** In situ XRD patterns [104]. Copyright 2022, Wiley–VCH GmbH

To ascertain the viability of bismuth halide as an anode for PIBs, a flower-like composite BiOBr_0.5_Cl_0.5_/rGO was synthesized to meet challenges of low stability and electronic conductivity of BiOX structure [106]. The 3D nanoflower was consisted of 2D nanosheets with the alternating arrangement of Br and Cl atoms, which is related to the self-hybridization of Br and Cl atomic orbitals. BiOBr_0.5_Cl_0.5_ was further coated with rGO to increase the electrical conductivity and K^+^ insertion space (Fig. 15a). Its unique self-hybridized structure enhances K^+^ adsorption and diffusion, leading BiOBr_0.5_Cl_0.5_/rGO to exhibit high capacity and long cycle life at an ultra-large current density (Fig. 15b, c). The in situ XRD reveals that BiOBr_0.5_Cl_0.5_/rGO stores potassium through intermediate state and alloying reaction (Fig. 15d, e). It is worth noting that the intermediate product K_x_BiOBr_0.5_Cl_0.5_ promotes a sustained release of Bi metal, which provides theoretical references for developing high-performance BiOX. In addition to structural engineering, research into the nanosize effect is also a popular avenue of investigation for modified bismuth halides, particularly given the difficulty in preparing bismuth halide grains smaller than 10 nm. Nevertheless, the investigation of the function of ultrasmall bismuth halide nanocrystalline anodes has been spearheaded in PIBs [104]. The synthesis of BiOCl crystals of only 6 nm in size, uniformly dispersed on reduced graphene oxide (RGO) nanosheets, was achieved by employing a nanorestriction strategy in lieu of the conventional solvent-thermal method (Fig. 16a). This strategy is also applicable to the synthesis of BiOBr and BiOI. The TEM analysis in Fig. 16b, c revealed that the average size and distribution of S-BiOCl-RGO exhibited no aggregation. The strong synergistic effect of conductive RGO and ultra-small BiOCl results in S-BiOCl-RGO exhibiting a high reversible capacity and a long cycling life (Fig. 16d, e). After cycling for over 10 months, 497 mAh g⁻^1^ remains available with a capacity retention of 94.3%. The potassium storage mechanism of S-BiOCl-RGO shown in Fig. 16f is consistent with the general characteristics of bismuth halides, involving a reversible phase transition process of BiOCl ↔ Bi ↔ K_3_Bi, with the intermediate phases being Bi, KCl and K_2_O generated by the reaction of K and BiOCl.

In addition to the common BiOCl matrix, BiOBr and BiOI systems have been reported for applications in alkali metal ion batteries. The electrochemical performances of BiOBr and BiOI materials are far inferior to that of BiOCl yet [13]. Consequently, BiOCl_0.5_Br_0.5_ and BiOBr_0.5_I_0.5_ were developed as a means of modifying bismuth-based halogen oxides. This was achieved by capitalizing on the strong built-in electric field formed between the two halogen atoms and the large layer spacing, which resulted in a notable enhancement in the electronic conductivity and potassium storage stability of the electrode materials [107, 108]. Furthermore, the construction of novel van der Waals heterostructures represents another avenue for modifying halogen oxides, which may prove advantageous for reaction kinetics. Examples of such structures include Bi_0.51_Sb_0.49_OCl/rGO, BiOCl/Ti_3_C_2_T_x_ MXene and BiOCl/amorphous antimony oxide (BOC/AAO) [105, 113].

All developed BiOX anode materials for PIBs are listed below (Table 6). Compared with pure Bi powder electrode, BiOX has obtained higher specific capacity. However, the reversible specific capacity of BiOX serial anodes is still far from the theoretical specific capacity (618 mA g^−1^) and cycling stability is poor, which requires further investigation [114]. The performance of BiOX anode materials is also strongly related to synthesis methods. Majority of the frequently used methods are likely to cause serious pollution and the process of preparation is complex. For instance, the 2D Bi_x_O_y_Cl_z_ nanosheets usually grow in strong alkaline environments, but the use of strong bases or acids is eco-unfriendly [116]. As a consequence, it is necessary to develop new green and simple synthesis routes to achieve efficient batch production, with the size and morphology of BiOX particle can be controlled. In addition, the promising directions for modifying BiOX anode materials in the future include the use of surface engineering, defect engineering, elemental engineering, heterostructure construction, increasing layer spacing, doping with transition metals or non-metallic elements and preparing BiOX grains with ultra-small nanometer size (< 6 nm) [117, 118].

**Table 6:** Performance comparison of BiOX

BiOX anode of PIBs				
Materials	Rate capability	Cycle capability	ICE (%)	Refs
BiOCl@N-CNTs	452 mAh g^–1^ at 0.1 A g^–1^; 118 mAh g^–1^ at 5 A g^–1^	156 mAh g^–1^ after 2000 cycles at 1 A g^–1^	–	[109]
BiOCl/Ti_3_C_2_T_x_	85 mAh g^–1^ at 0.5 A g^–1^(DME–KFSI-5)	183 mAh g^–1^ after 100 cycles at 0.05 A g^–1^(DME–KFSI-5)	50	[110]
BiOCl/G	130 mAh g^–1^ at 0.5 A g^–1^	251 mAh g^–1^ after 50 cycles at 0.05 A g^–1^	–	[111]
BiOCl NFAs	346 mAh g^–1^ at 0.05 A g^–1^; 175 mAh g^–1^ at 1 A g^–1^	213 mAh g^–1^ after 50 cycles at 0.05 A g^–1^	43.5	[112]
S-BiOCl-RGO	521 mAh g^–1^ at 0.05 A g^–1^; 205 mAh g^–1^ at 5 A g^–1^	194 mAh g^–1^ after 3000 cycles at 5 A g^–1^	76.5	[104]
BiOCl	–	232 mAh g^–1^ after 1000 cycles at 0.5 A g^–1^	–	[113]
1-BOC/3-AAO	395.9 mAh g^–1^ at 0.5 A g^–1^	316.9 mAh g^–1^ after 800 cycles at 0.5 A g^–1^	–	[105]
BBNs	534.1 mAh g^–1^ at 0.5 A g^–1^; 420 mAh g^–1^ at 10 A g^–1^	198 mAh g^–1^ after 1500 cycles at 10 A g^–1^	80.4	[114]
BiOCl	> 200 mAh g^–1^ at 1 A g^–1^	151.8 mAh g^–1^ after 100 cycles at 0.05 A g^–1^	–	[115]
Bi_0.51_Sb_0.49_OCl/rGO	407 mAh g^–1^ at 0.1 A g^–1^; 319 mAh g^–1^ at 1 A g^–1^	360 mAh g^–1^ after 1000 cycles at 0.1 A g^–1^	–	[101]
BiOBr_0.5_Cl_0.5_/rGO	349.6 mAh g^–1^ at 0.5 A g^–1^; 246.4 mAh g^–1^ at 50 A g^–1^	218.1 mAh g^–1^ after 2400 cycles at 5 A g^–1^	80.6	[106]

### Other Bismuth-Based Materials

**Fig. 17:**
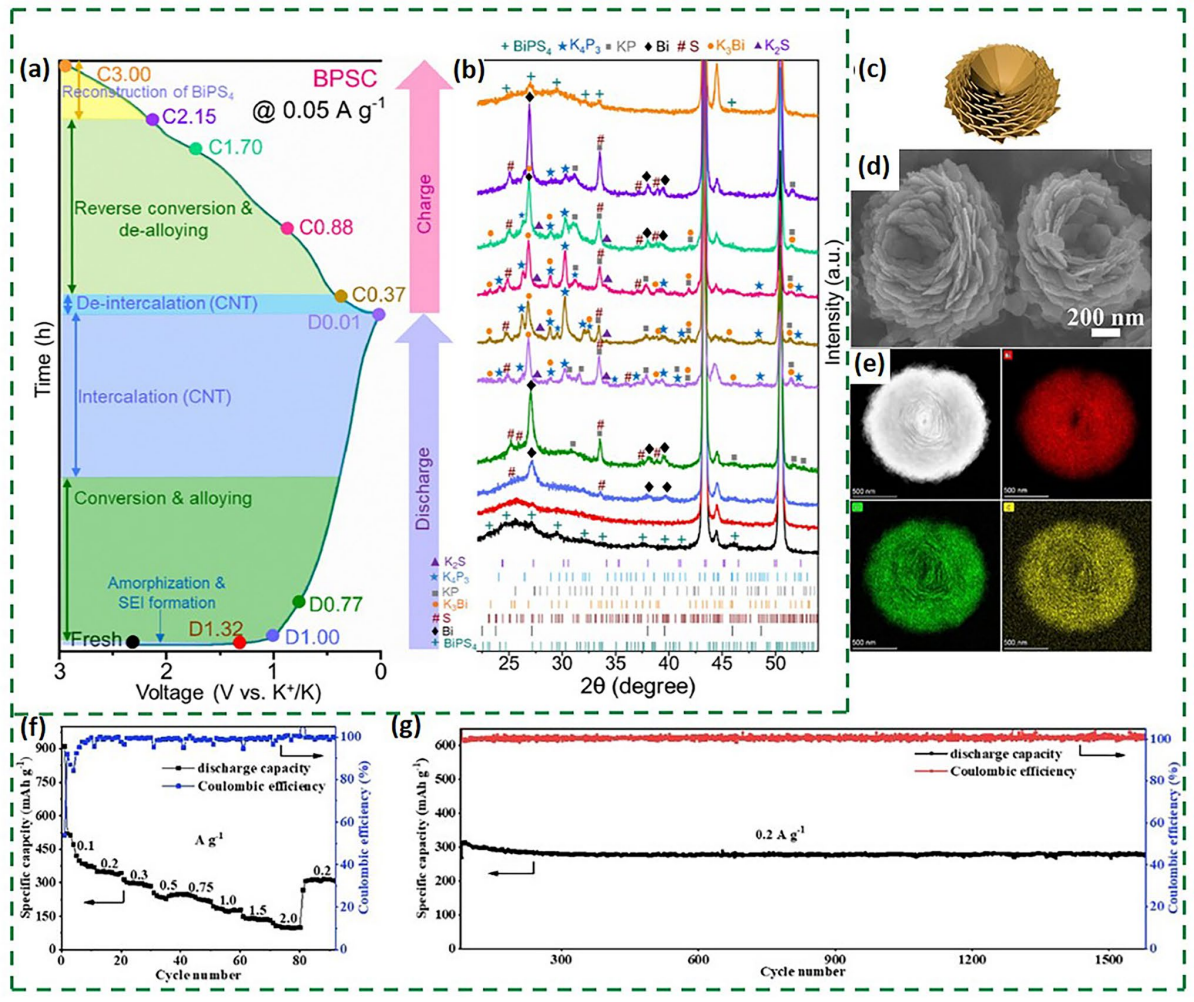
**a** First cycle charge/discharge profile of BPSC (using the KFSI electrolyte) tested at 0.05 A g^−1^ with selected potentials for the HRXRD analyses presented in **b** [121]. Copyright 2021, Elsevier B.V. **c–e** Demonstration, a typical SEM image and HAADF-STEM image of the obtained BCO, respectively. **f** Rate performance of BCO-rGO (1–1) electrode. **g** Prolonged cycling performance of BCO-rGO (1–1) electrode after rate measurement in **f** [122]. Copyright 2021, Wiley–VCH GmbH

Other unique materials with special potassium storage mechanisms were developed from different perspectives. For example, metal sulfides and phosphides are feasible anodes for PIBs due to the reactions between S/P and K^+^ [119, 120]. A tunnel-structured BiPS_4_ and CNT composite (BPSC) was presented [121] as anode for PIBs to simultaneously exploit the synergic advantages of bismuth sulfides and phosphides (Fig. 17a, b). BPSC delivered remarkable electrochemical performance due to multiplex reversible conversion and alloying reactions, which can be described as BiPS_4_ ← Charge/Discharge → K_4_P_3_ + KP + K_3_Bi + K_2_S. Therefore, designing special structure can provide abundant ion and electron transport paths for large rate capability and good cycling performance. In addition to this, the interfacial chemistry is considered to be an important influencing factor for potassium-ion storage, which is related to the electrolyte and electrode materials. There have been efforts in this direction such as the innovatively proposed two-dimensional (BiO)_2_CO_3_(BCO) [122]. The hierarchical BCO was presented flower-like morphology and integrated by 2D nanosheets (Fig. 17c-e). BCO compounded with reduced graphene oxide (rGO) electrode-delivered capacity of 450 mAh g^−1^ at 0.1 A g^−1^ and its long-term cycling performance was also superb as BCO-rGO decayed rate of only 0.007% after over 1500 cycles (Fig. 17f, g). It is necessary to create more novel anode architectures for higher performed PIBs on the basis of understanding the interplay of bismuth-based materials and electrolytes.

**Fig. 18:**
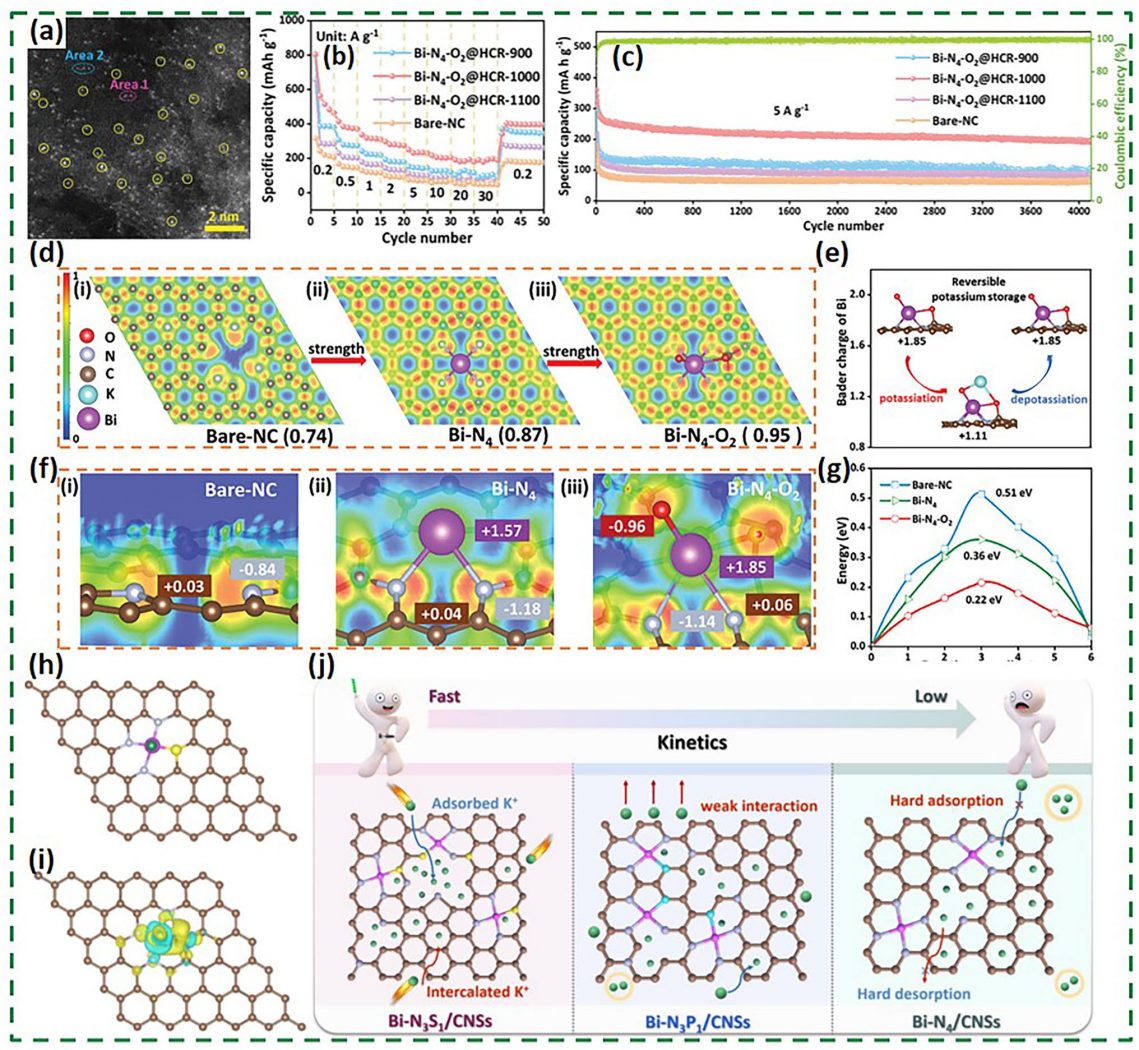
**a** Atomic resolution HAADF-STEM images of Bi-N_4_-O_2_@HCR-1000. **b**, **c** Rate performances and cycle capabilities at 5 A g^−1^ of Bi-N_4_-O_2_@HCR-900, Bi-N_4_-O_2_@HCR-1000, Bi-N_4_-O_2_@HCR-1100 and Bare-NC. **d** Electron localization function of Bare-NC, Bi-N_4_ and Bi-N_4_-O_2_ structures. **e** Bader charge value of Bi atom during adsorption and desorption. **f** Bader charge values. **g** Energy barriers for K^+^ diffusion [123]. Copyright 2024, Wiley–VCH GmbH. **h**, **i** Top views of K atom adsorption model and charge density difference in Bi-N_3_S_1_/CNSs. **j** Schematic illustration of the K^+^ transport and adsorption on Bi-N_3_S_1_/CNSs, Bi-N_3_P_1_/CNSs and Bi-N_4_/CNSs, respectively [124]. Copyright 2024, ELSEVIER B.V. and Science Press

In the field of bismuth-based materials, our research group has successfully synthesized bismuth metal at the single-atom level and demonstrated its potential as an anode material for PIBs. Specifically, the nitrogen and oxygen double-coordinated bismuth metal atomic sites were abundantly anchored on honeycomb carbon nanorods (Fig. 18a). It results in the formation of Bi–N_4_–O_2_@HCR materials with a capacity of 190.7 mAh g^−1^ at 30 A g^−1^ and over 4200 cycles at 5 A g^−1^ (Fig. 18b, c) that exhibit a combination of the high capacity of bismuth metal and the stability of carbon materials [123]. DFT calculations further reveal the relationship between the coordination structure of Bi–N_4_–O_2_ and the storage of K^+^. The Bi–N_4_–O_2_ coordination configuration shows the highest ELF value, corresponding to the strongest electron localization function (Fig. 18d). The Bader charge reversible change of Bi atoms in Bi–N_4_–O_2_ corresponds to reversible potassium storage (Fig. 18e, f). In addition, the coordination of O and N atoms enhances charge transfer and ionic diffusion so that Bi–N_4_–O_2_ can obtain the lowest K^+^ migration energy barrier (Fig. 18g). This unique metal atom coordination concept in PIBs was successfully validated. On the basis of this, we also explored [124] the impacts of bismuth metal coordination environments. The Bi–N_3_S_1_ coordination configuration exhibited the lowest adsorption energy and strongest charge density difference (Fig. 18h, i), which anchored on carbon nanosheets (Bi–N_3_S_1_/CNSs) can produce abundant active sites for rapid kinetics of potassium storage (Fig. 18g). These works provide new insights into the study of bismuth and carbon anodes in PIBs. Our work has initiated new avenues for the advancement of bismuth- and carbon-based materials and our team is pursuing this direction, including investigating the effects of bismuth-based bimetallic single atoms and even bismuth-based bionanomaterials on the anode for PIBs. Except the bismuth-based materials we mentioned above, other original bismuth-based anodes for PIBs have also been studied (Table 7).

**Table 7:** Performance comparison of other bismuth-based anodes

Other bismuth-based anodes of PIBs				
Materials	Rate capability	Cycle capability	ICE (%)	Refs
BPSC	863 mAh g^–1^ at 0.05 A g^–1^; 498 mAh g^–1^ at 2 A g^–1^	489 mAh g^–1^ after 600 cycles at 1 A g^–1^	81.9	[121]
BCO-rGO (1–1)	450 mAh g^–1^ at 0.1 A g^–1^; 100 mAh g^–1^ at 2 A g^–1^	ca. 300 mAh g^–1^ after 1500 cycles at 0.2 A g^–1^	54	[122]
(Bi_0.47_Sb_0.53_)PO_4_/G	426 mAh g^–1^ at 0.05 A g^–1^; 297 mAh g^–1^ at 0.5 A g^–1^	288 mAh g^–1^ after 400 cycles at 0.5 A g^–1^	53.6	[125]
AgBiS_2_ NCs	420 mAh g^–1^ at 0.5 A g^–1^; 210 mAh g^–1^ at 5 A g^–1^	over 300 mAh g^–1^ after 300 cycles at 2 A g^–1^	–	[126]
MP Bi-Cu_2-*x*_S	290 mAh g^–1^ at 0.05 A g^–1^; 135 mAh g–1 at 0.8 A g^–1^	170 mAh g^–1^ after 200 cycles at 0.1 A g^–1^	28.2	[127]
BiPO_4_/SP	401.1 mAh g^–1^ at 0.02 A g^–1^; 97.1 mAh g^–1^ at 0.5 A g^–1^	116 mAh g^–1^ after 100 cycles at 0.2 A g^–1^	29.8	[128]
Cu_3_BiS_3_	426.8 mAh g^–1^ at 0.1 A g^–1^; 58.3 mAh g^–1^ at 10 A g^–1^	158.8 mAh g^–1^ after 2000 cycles at 0.5 A g^–1^	69.8	[129]
H-Bi-Ti-EG	400 mAh g^–1^ at 0.1 A g^–1^; 280 mAh g^–1^ at 1 A g^–1^	85.8 mAh g^–1^ after 6000 cycles at 2 A g^–1^	55.2	[130]
Bi-N_4_-O_2_@HCR-1000	563.7 mAh g^–1^ at 0.2 A g^–1^; 190.7 mAh g^–1^ at 30 A g^–1^	192.2 mAh g^–1^ after 4200 cycles at 5 A g^–1^	52.1	[123]
Bi-N_3_S_1_/CNSs	447.9 mAh g^–1^ at 0.2 A g^–1^; 129.2 mAh g^–1^ at 10 A g^–1^	203.3 mAh g^–1^ after 5000 cycles at 5 A g^–1^	42.1	[124]

## Summary

In this review, we comprehensively summarized the advances on bismuth-based anode materials of PIBs, including metallic Bi, bismuth oxides, bismuth chalcogenides, alloys, bimetallic oxides, bismuth oxyhalides, etc. We not only discuss the potential of bismuth-based materials as precursors to derive new materials, but also illustrate the novel design of introducing single Bi metal atoms on carbon carriers to take advantage of both bismuth- and carbon-based materials. We present the basic properties, working principles, advantages and disadvantages of all bismuth-based materials. We emphasized on the potassium-ion storage mechanisms of various bismuth-based anode materials and the influence of their morphologies and structures on the electrochemical performance for PIBs, involving multiple characterization techniques and theoretical analysis. It is worth noting that the potassium storage mechanisms of total bismuth-based anode materials are partially similar, which all involve reversible alloying reactions Bi ↔ K_3_Bi. In addition to the unique potassium storage mechanism, high theoretical capacity and safe operating voltage are prerequisites for bismuth-based materials as potential anodes [12]. Furthermore, the conductivity of metal-based materials is more competitive compared to other materials. Although bismuth-based anode materials have great potential and promising future in potassium storage, their production costs are extremely high due to the scarcity of bismuth resources in the earth's crust [131]. In addition, the strong volume expansion generated during cycles leads to the destruction of material structures and rapid capacity decay. These rather tricky issues cause bismuth-based anode materials to face many challenges on practical applications. Therefore, many strategies that have been reported to optimize the electrochemical performance of bismuth-based anodes on potassium-ion storage include constructing carbon materials coating, superficial chemical engineering, alloying and nanoengineering structures [132].

## Outlook

**Fig. 19:**
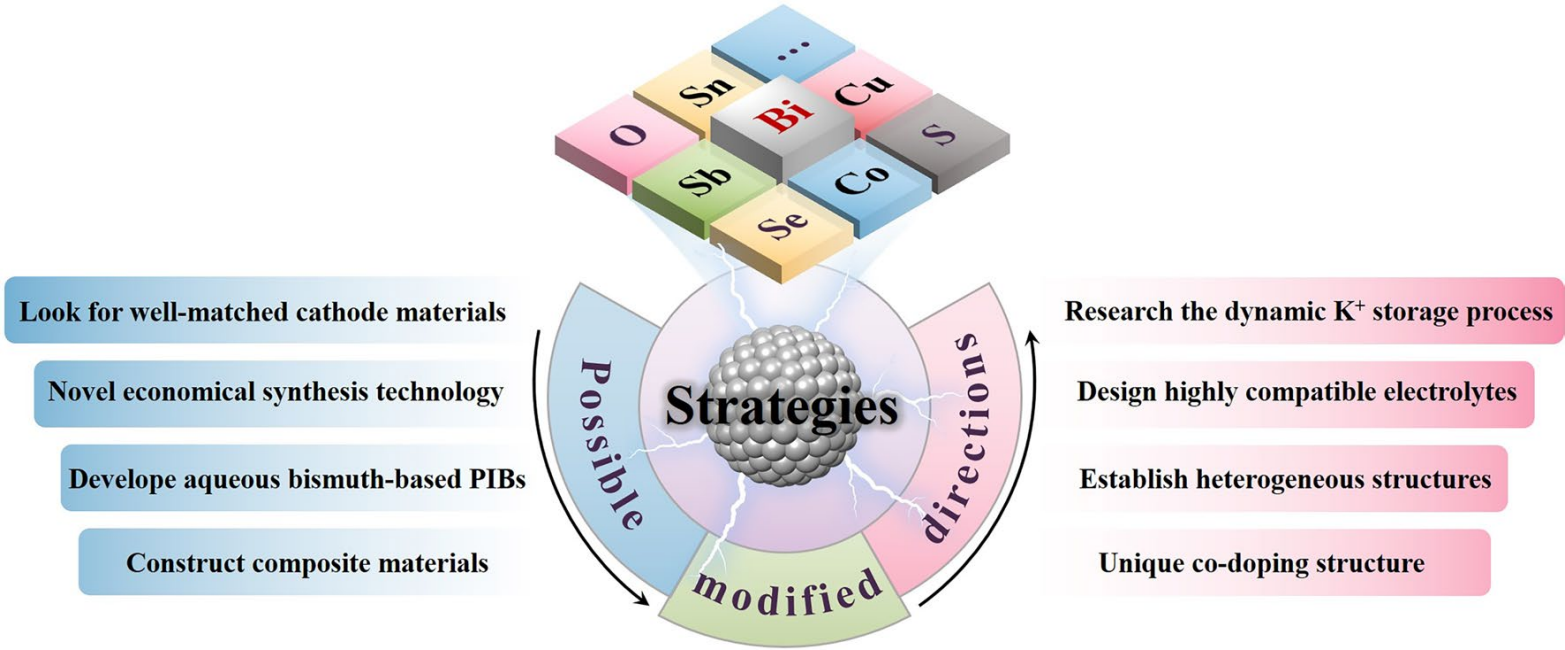
Possible modification directions of bismuth-based anode materials

Above means have been effective in mitigating the volumetric stress on active materials. However, these approaches deserve further discussions and new modification methods need to be explored to promote the development of PIBs. It is striving for achieving the short-term target that PIBs exhibit the energy density comparable to LIBs in the future, while the cycling performance of PIBs can catch up with LIBs and SIBs at the material level. Then, PIBs truly become an effective technology that can be a supplement to LIBs and SIBs at an early date. The development of power batteries requires comprehensive consideration involving high energy and power densities, long cycle life and high security. To achieve these desired performances simultaneously, it is necessary to explore promising materials for commercialized PIBs based on the four components of PIBs. For cathode materials that determine the maximum discharge voltage of the full cell, the layered metal oxides are currently more promising. The lack of suitable anode materials is also a factor hindering the rapid development of PIBs, and bismuth-based and carbon-based anodes are advantageous in terms of overall performance and cost-effectiveness. In addition, high-quality electrolytes and separators are critical to improving the energy density and stability of PIBs. The more potential electrolytes are mainly ester and ether groups to solve the dendrite problem, while the separator is developing in the direction of composite separator. We believe that the possible key directions shown in Fig. 19 for future researches may focus on, but are not limited to, the followings:

1. It is well known that bismuth-based materials can form composites with carbon, yet their energy density is affected by the type of carbon materials, including hard carbon, soft carbon, graphite, graphene, carbon nanotubes, etc. The morphology and structure of carbon can influence the properties of composite through generating different specific surface areas and active sites. For example, researchers use single-doped, double-doped or triple-doped carbon materials with elements such as N, P and S to compound with bismuth-based materials for the structural modification. *N*-doping can facilitate fast K^+^ diffusion and surface wettability, while *P*-doping and *S*-doping can effectively improve hydrophilicity and conductivity [133]. Besides, it is possible to consider combining bismuth-based with biochar which is no need for high-temperature carbonization, so that the energy consumption and environmental pollution can be effectively reduced. Bismuth-based biochar materials hold promise for energy transitions. It is worth noting that the additive amount of carbon materials is also critical for performances improvement. Therefore, it is quite crucial to select appropriate carbon materials and additive amount for balancing the performances. In addition, it is recommended that people study on the combination of bismuth-based materials with chalcogenides (S, Se) or multiple components for PIBs in depth [134, 135].
2. According to few relevant literatures, the bismuth base can enter the composite carrier in the form of metal-doped atom, achieving the co-doping of metallic and non-metallic atoms. The coordination environment of central metal atom has an effect on potassium storage, such as the degree of electron asymmetry distribution. The strong electronic localization leads to charge accumulation and elimination, which in turn accelerates charge transfer and reduces the ionic diffusion energy barrier. Meanwhile, the co-doping sites can decrease K^+^ adsorption energy and expand the layer spacing of conductive matrix, so that the surface adsorption and ionic diffusion are enhanced together. This will provide abundant defects as potassium-ion storage active sites and fast reaction kinetics. This is one of the most promising modification methods for bismuth-based anode materials. Moreover, except for the basic rate and cycling performance, PIBs with the wide temperature domain are worth exploring, especially the fast potassium storage performance of bismuth-based composite anode materials at an extremely low temperature.
3. Although bismuth-based anode materials can be successfully synthesized in the laboratories, it is very difficult to produce materials with controlled structures and morphologies in industrial mass especially composites. Besides, the preparation process can generate serious pollution. Hence, it is crucial to explore simple, green, safe and cost-effective synthesis methods, such as pulsed laser irradiation techniques. There are some novel techniques such as plasma ball milling and cutting, which can help us prepare materials quickly and without contamination. Bismuth-based nanoparticles with low profile are less prone to agglomerate during cycling, which can reduce bulk stress so that it is one of the most feasible directions to prepare ultra-small bismuth-based anode nanomaterials.
4. With regard to the same electrode materials, the electrochemical performances are entirely different by using different electrolytes [136]. Currently, electrolytes for PIBs face some critical issues, such as the poor safety of organic electrolytes, the small voltage window of aqueous electrolytes, the relatively low ionic conductivity of solid electrolytes and the high cost of ionic liquid electrolytes. Therefore, we need to design highly compatible safe electrolytes and additives, to form a series of stable, resilient and thin solid electrolyte interphase, and to suppress side reactions at electrode/electrolyte interfaces. Some possible strategies include regulating the concentration of electrolytes, designing lightweight and high-modulus solid electrolytes and exploring new potassium salts, solvents and additives. For bismuth-based anode materials with the dual mechanism of conversion and alloying reactions, it is also necessary to prevent the intermediate products of conversion reactions from dissolving.
5. Compared to traditional organic electrolyte solvents, aqueous batteries that use water as the solvent are safer and more environmentally friendly. The development of aqueous bismuth-based PIBs has great potential due to the low toxicity and high capacity of bismuth-based materials. The common water-soluble potassium salt electrolytes face the problem of narrow electrochemical window. Therefore, designing new materials and optimizing aqueous electrolytes are feasible strategies to achieve aqueous bismuth-based PIBs with high energy density and cycling stability. These include that using water-in-salt concentrated electrolyte to widen the window, compounding water-soluble potassium salts and organic additives and attempting hydrogel electrolyte to improve the electrochemical stability of aqueous bismuth-based PIBs.
6. To both promote rapid potassium-ion transport and prevent agglomeration of active materials, we can construct heterogeneous structures especially bismuth-based chalcogenides anode materials, increase the contact area of electrode/electrolyte and widen the layer spacing of bismuth-based anode materials. These strategies can also counteract the volume expansion and structural changes during the delamination and embedding process of potassium ion. The built-in electric field effects of heterostructures can also significantly improve the electrical conductivity of semi-conductive BiOX anode materials [13].
7. It is viable to research the mechanism of potassium-ion storage in bismuth-based anode materials, the formation of SEI, the diffusion of potassium-ion and detailed electron transport process in depth by using advanced in situ techniques, theoretical calculations and experimental simulations. In situ/ex situ Raman and XRD coupled with theoretical calculations are advanced methods to probe the potassium storage mechanism of bismuth-based materials at present. The formation and accumulation mechanisms of SEI can be probed by advanced techniques such as nuclear magnetic resonance (NMR). Besides, in situ TEM is also a crucial characterization technique to monitor the structural phase transition and morphological change of bismuth-based anode materials in real time during their charging and discharging processes, thus dynamically revealing the storage mechanism and size effect of K^+^ at the atomic scale. These will provide the theoretical bases for optimizing the K^+^ storage performances of bismuth-based anode materials.
8. It is necessary to seek suitable commercial cathode materials to match bismuth-based anode materials, so as to maximize the excellent performances of bismuth-based anodes and develop full cells with high capacity of PIBs. In order to enhance the matched-degree between bismuth-based anode and cathode materials, we need to take into account not only the structural compatibility but also the interface of electrolyte and electrode. For instance, it is quite important to calculate a reasonable ratio factor of cathode and anode capacity. When using bismuth-based anode materials with poor structural stability, the ratio of anode capacity to cathode capacity may be considered to exceed slightly 1.0, which can improve the cycle performance of potassium-ion full cell. Besides, the SEI layer on anode and the CEI layer on cathode can be synergistically modulated by selecting suitable electrolyte system to build high-performance PIBs [21]. Among them, bismuth-based anode and organic cathode materials are expected to realize low-cost PIBs.

Finally, we should consider comprehensively and favorably coordinate various modification methods to improve the reversible capacity of bismuth-based anodes while optimizing the cycling stability. At present, the advance of bismuth-based anodes for PIBs is still in the preliminary stage. Therefore, it is crucial to coordinate the relationship of structures and performances of bismuth-based materials. The challenge we must overcome is how to develop efficient synthesis methods and elaborately design structures of materials before commercialization of bismuth-based anodes. Finally, it is sincerely hoped that this review will provide reading public with references on the applications of bismuth-based anode materials in PIBs or other power batteries.
